# Effectiveness of school food environment policies on children’s dietary behaviors: A systematic review and meta-analysis

**DOI:** 10.1371/journal.pone.0194555

**Published:** 2018-03-29

**Authors:** Renata Micha, Dimitra Karageorgou, Ioanna Bakogianni, Eirini Trichia, Laurie P. Whitsel, Mary Story, Jose L. Peñalvo, Dariush Mozaffarian

**Affiliations:** 1 Friedman School of Nutrition Science and Policy, Tufts University, Boston, MA, United States of America; 2 Department of Food Science and Human Nutrition, Agricultural University of Athens, Athens, Greece; 3 Policy Research, American Heart Association, Dallas, TX, United States of America; 4 Global Health Institute and Community and Family Medicine, Duke University, Durham, NC, United States of America; Universitat de Lleida-IRBLLEIDA, SPAIN

## Abstract

**Background:**

School food environment policies may be a critical tool to promote healthy diets in children, yet their effectiveness remains unclear.

**Objective:**

To systematically review and quantify the impact of school food environment policies on dietary habits, adiposity, and metabolic risk in children.

**Methods:**

We systematically searched online databases for randomized or quasi-experimental interventions assessing effects of school food environment policies on children’s dietary habits, adiposity, or metabolic risk factors. Data were extracted independently and in duplicate, and pooled using inverse-variance random-effects meta-analysis. Habitual (within+outside school) dietary intakes were the primary outcome. Heterogeneity was explored using meta-regression and subgroup analysis. Funnel plots, Begg’s and Egger’s test evaluated potential publication bias.

**Results:**

From 6,636 abstracts, 91 interventions (55 in US/Canada, 36 in Europe/New Zealand) were included, on direct provision of healthful foods/beverages (N = 39 studies), competitive food/beverage standards (N = 29), and school meal standards (N = 39) (some interventions assessed multiple policies). Direct provision policies, which largely targeted fruits and vegetables, increased consumption of fruits by 0.27 servings/d (n = 15 estimates (95%CI: 0.17, 0.36)) and combined fruits and vegetables by 0.28 servings/d (n = 16 (0.17, 0.40)); with a slight impact on vegetables (n = 11; 0.04 (0.01, 0.08)), and no effects on total calories (n = 6; -56 kcal/d (-174, 62)). In interventions targeting water, habitual intake was unchanged (n = 3; 0.33 glasses/d (-0.27, 0.93)). Competitive food/beverage standards reduced sugar-sweetened beverage intake by 0.18 servings/d (n = 3 (-0.31, -0.05)); and unhealthy snacks by 0.17 servings/d (n = 2 (-0.22, -0.13)), without effects on total calories (n = 5; -79 kcal/d (-179, 21)). School meal standards (mainly lunch) increased fruit intake (n = 2; 0.76 servings/d (0.37, 1.16)) and reduced total fat (-1.49%energy; n = 6 (-2.42, -0.57)), saturated fat (n = 4; -0.93%energy (-1.15, -0.70)) and sodium (n = 4; -170 mg/d (-242, -98)); but not total calories (n = 8; -38 kcal/d (-137, 62)). In 17 studies evaluating adiposity, significant decreases were generally not identified; few studies assessed metabolic factors (blood lipids/glucose/pressure), with mixed findings. Significant sources of heterogeneity or publication bias were not identified.

**Conclusions:**

Specific school food environment policies can improve targeted dietary behaviors; effects on adiposity and metabolic risk require further investigation. These findings inform ongoing policy discussions and debates on best practices to improve childhood dietary habits and health.

## Introduction

Diets of most children and adolescents (hereafter referred to as children) remain poor, with tremendous consequences for metabolic diseases, overweight and obesity, and other nutrition-related illness [1–4]. Childhood is also a critical period to establish lifelong eating habits which influence future risk of obesity and cardiometabolic diseases [5–7]. Youth consume between one-third to one-half of meals at school, making this a crucial setting for interventions that alter the food environment [8]. Considering that almost all children obtain some years of schooling, and of diverse ethnic and socio-economic groups, health promotion efforts in schools could have a broader impact on eating behaviors and future disease risk.

Promising school food environment policies include direct provision of healthful foods/beverages such as fruits and vegetables (F&V), quality standards for competitive foods and beverages (foods and beverages sold outside of school meal programs), and quality standards (targets for foods, nutrients/energy) for school meals (lunch, breakfast) [8]. For example, in 2008, a US Fresh Fruit and Vegetable Program (FFVP) was expanded nationally for elementary schools with highest low-income enrolments to provide free F&V to students outside usual school meals [9]; and in 2007, a similar free school fruit programme was implemented in Norway to provide daily a free piece of fruit or vegetable to all secondary school students [10]. The Healthy, Hunger-Free Kids Act in 2010 [11] introduced Smart Snack Standards for competitive foods and beverages in schools receiving federal meal funding, including restriction of sugar-sweetened beverages (SSBs) to be fully implemented by 2014–15 [12]. In 2012, US National School Lunch and School Breakfast Programs nutrition standards were significantly updated to be more consistent with US Dietary Guidelines [13], and in 2015 the UK Department of Education mandated revised standards for all food served in schools [14].

Yet, effectiveness of these food environment policies for improving children’s habitual dietary habits, adiposity, or metabolic risk is not well-established. Understanding these effects is critical to estimate benefits of existing programs as well as need for their expansion; and to elucidate potential harms from their elimination as suggested by potential new federal priorities in the US [15,16]. Prior studies have reviewed whether a range of school dietary interventions increase F&V consumption but often without focusing on environmental policies [17–22]; while other systematic reviews have been qualitative [23], assessed efficacy of competitive food/beverage standards informed mainly by cross-sectional studies [24], or focused on educational (rather than environmental) interventions [25]. Other reviews have grouped together highly varied programs, e.g., teacher training, child education, family components, labeling, pricing changes, behavioral techniques, and school gardens [26–32]. Thus, effectiveness of school food environment policies remain unclear, including potential differences for in-school vs. habitual (within and outside school) intakes. To address these gaps in knowledge, we systematically investigated and quantified the effects of school food environment interventions -carefully exploring sources of heterogeneity-, including provision of healthful foods/beverages, competitive food/beverage standards, and school meal standards, on habitual and in-school dietary consumption, adiposity, and metabolic risk factors in children. This investigation was performed as part of the Food-PRICE (Policy Review and Intervention Cost-Effectiveness) Project (www.food-price.org).

## Methods

PRISMA recommendations were followed throughout all stages of this meta-analysis (Appendix A in S1 File) [33]. The objective, search strategy, and selection criteria were specified in advance (Appendix B in S1 File).

### Primary exposures and outcomes

The primary intervention was school food environment policies targeting food/beverage availability across the school setting (e.g., classroom, cafeterias, vending machines, tuck shops) including direct provision (free, reduced-price, or full-price) of healthful foods or beverages outside of usual school meals (e.g., fresh F&V programs, water fountains, increased availability of healthy foods at vending machines), nutritional quality standards for competitive foods/ beverages, and nutritional quality standards for school meals (lunch, breakfast). The primary outcome was the change in habitual consumption of the targeted food, beverage, or nutrient, evaluated by reported intakes or objective sales/purchases data as a proxy for consumption. Secondary outcomes included changes in in-school meal nutrient content and intake (to compare and contrast to findings for habitual intake), total caloric intake, adiposity (body mass index (BMI), prevalence of overweight (≥85^th^-95^th^ percentile), obesity (≥95^th^ percentile) or overweight/obesity combined); and metabolic measures (e.g., blood lipids, blood glucose, blood pressure).

### Search strategy

Multiple online databases were systematically searched including PubMed, EconLit, CINAHL, CABI, Web of Science, PAIS, Cochrane Library, AGRIS, Open Grey, Faculty of 1000 and EMBASE earliest available through March 9, 2014 without restrictions on language or country. Online searches were updated in PubMed from March 10, 2014 to December 14, 2017 as this is the primary database for research in this field, and the majority (>95%) of relevant papers in the initial review were identified in PubMed. The intervention periods of identified publications largely preceded widespread implementation of the new US school lunch standards, Smart Snacks Standards, FFVP, or revised UK school meal standards. Search terms utilized 4 categories, including on the intervention, dietary target, outcome, and setting (Appendix C in S1 File); supplemented by hand-searching of citations and the first 20 “related articles” in PubMed for each final included article. Titles/abstracts were screened by one investigator; and for all potentially relevant articles, full-texts were retrieved.

### Study selection

Full-text manuscripts were evaluated independently and in duplicate, with differences resolved by consensus or, if necessary, group discussion. Inclusion criteria were (a) all randomized or quasi-experimental interventions that (b) assessed the impact of school food environment policies in preschool, primary, or secondary schools on the outcomes of interest among generally healthy children age 2–18y; and (c) reported a quantitative change in the outcome (Appendix B in S1 File). We excluded cross-sectional, retrospective, case-control, modeling, methodology, and laboratory studies; reviews, commentaries, books, and studies for which full-text articles could not be retrieved. Studies were excluded if the policy focused on changes outside of food/beverage availability (e.g., student education, food labeling, price changes), if the food/beverage environmental policy was a minor component (qualitatively, <30%, as judged by two independent reviewers) of a multi-component intervention, if intervention duration was <4 weeks, or if only knowledge or attitudes were evaluated as outcomes.

### Data extraction

Data were extracted independently and in duplicate using standardized electronic templates (Microsoft Access, Office 2010). Extracted information included first author, publication year, study location, design, population (age, sex, race, sample size), intervention characteristics (components, targets, duration), outcome data including habitual (within and outside school) and in-school (e.g., lunch, breakfast, total in-school) intakes (definition, ascertainment methods, effect size, precision estimate), covariates, and for multi-component interventions, the relative contribution of the food environment policy component to the overall intervention (low: 30–59%, medium: 60–89%, high: ≥90%; qualitatively assessed independently and in duplicate). Missing data or definitions were resolved by direct author contact, where possible.

For outcomes evaluated at multiple time-points, we extracted the latest follow-up measure at end-intervention. Sustainability findings based on follow-up after end-intervention were also extracted when available and ≥4 weeks duration. Study quality was assessed independently and in duplicate based on study design, assessment of exposure, assessment of outcome, control for confounding, and evidence of selection bias (Table A in S1 File). Differences in data extraction and quality assessment between investigators were infrequent (concordance >95%) and resolved by consensus.

### Statistical analysis

Analyses were conducted using STATA14 (College Station, TX: StataCorp LP). For each policy, study-specific effect sizes were pooled using inverse-variance random-effects meta-analysis. For interventions with an external control group, we evaluated between-group continuous changes at follow-up, adjusted for baseline values and relevant covariates; for quasi-experimental studies with no control group, we evaluated within-group changes [34]. Statistical uncertainty (standard error, SE) was extracted or calculated based on other statistics (Appendix D in S1 File). For paired observations without reported covariance, we used a correlation of 0.5 for main analysis and 0.1 and 0.9 for sensitivity analyses [34]. In addition to continuous effect sizes, we extracted other relevant effect sizes (e.g., percentage meeting a cutpoint, odds ratio, ratio of the means, other relative changes) and their statistical uncertainty. Separate intervention arms or outcomes from the same study were included as separate estimates in the meta-analyses; subgroup findings from the same intervention arm or outcome (e.g., by sex, age) were first combined using study-specific meta-analysis.

We separately pooled findings for direct provision of healthful foods and beverages, competitive foods and beverage standards, and school meal standards. Effect sizes were standardized to consistent units: e.g., 80 g serving/d for F&V, 12-oz serving/d for SSBs, 8-oz serving (glass)/d for water, kcal/d for calories, % energy (E)/d, g/d or mg/d for nutrients, and kg/m^2^ or z-score for BMI. Endpoints that could not be standardized (e.g., consumption expressed as a score, proportion of children consuming a given level) or separately meta-analyzed were included in qualitative assessment of the evidence. When multiple overlapping outcomes were reported (e.g., fruit with vs. without 100% juice), we extracted the outcome mostly closely aligned to a standardized definition, e.g. total fruits (fresh, raw, canned, or dried), excluding fruit juice; total vegetables, excluding white potatoes; and total SSBs (soda, energy drinks, sweetened teas, etc.). For studies reporting subcomponents of these definitions (e.g., separate subtypes of vegetables, of sweet snacks, F&V separately), we first summed these subtypes.

Cochran's Q and I^2^ statistics assessed between-study heterogeneity [35]. Meta-regression and stratified/subgroup meta-analysis explored potential prespecified heterogeneity sources when at least 5 study estimates were present, including design (randomized, quasi-experimental), region (US/Canada, Europe/New Zealand), intervention level (national, statewide, citywide, local), executing agent (law, governmental policy, program), components (food environment policy only, multi-component), follow-up duration (≥ or <median), school level (preschool, primary, secondary, mixed), school type (public, private, mixed), outcome definition (primary, alternative), relative contribution of the food environment policy to the overall intervention (low: 30–59%, medium: 60–89%, high: ≥90%), type of provision (free vs. reduced/full cost; direct vs. indirect), targeted caloric intake (yes, no), outcome being a primary or secondary study endpoint, and study quality score (0–3, 4–5). Potential publication bias was assessed visually using funnel plots and statistically by Egger's and Begg's tests [36].

## Results

### Study characteristics

**Fig 1:**
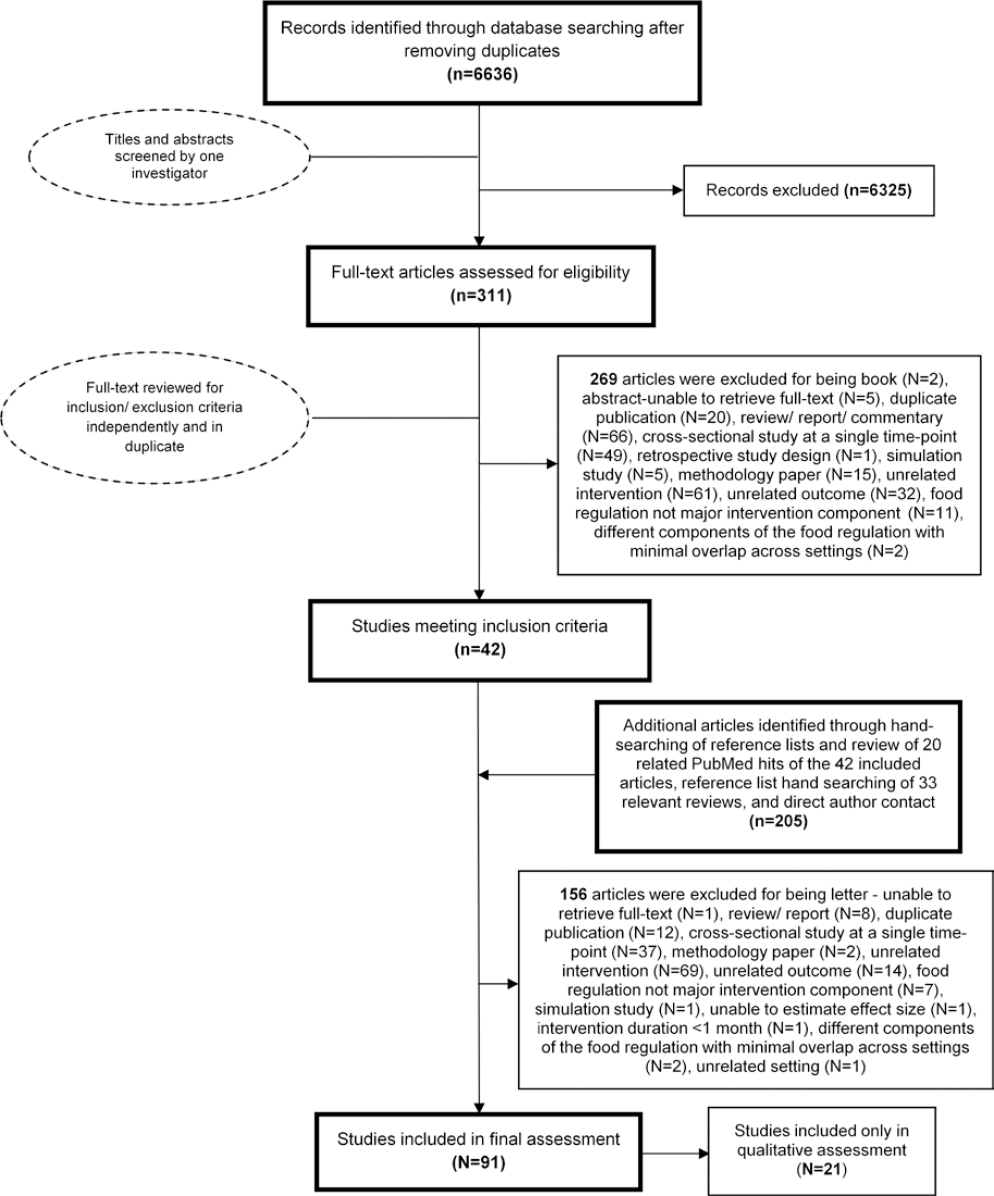
Screening and selection process of interventions evaluating the impact of school food environment policies on dietary habits, adiposity, or metabolic risk factors in children.

Of 6,636 identified articles, 91 interventions met inclusion criteria, including 39 randomized and 52 nonrandomized studies evaluating 1 or more food environment policy strategy (Fig 1, Table 1). These included direct provision of healthful foods/beverages (N = 40) [10,37–75], competitive food/beverage standards (N = 29) [66–72,74–95], and school meal standards (N = 39) [73–75,90–126]. Most studies were conducted in the US (N = 55), followed by the UK (N = 11), Netherlands (N = 7), Norway (N = 6), Canada (N = 3), South Korea (N = 2) and others (N = 1 each). About half of interventions (N = 49, 54%) were multi-component, with the relative contribution of the food environment policy component ranging from 30–100%. Data on race, socioeconomics, response rate, and urban/rural setting were largely not reported. Longest follow-up was 47 months in randomized and 60 months in quasi-experimental interventions. Forty-seven intervention studies were in primary schools, 27 in secondary schools, 1 in preschool, and 13 in mixed schools; 1 did not specify. Two studies reported only sustainability effects. Given types of outcomes reported, 21 studies were only included in qualitative assessment.

**Table 1:** Identified randomized and quasi-experimental interventions evaluating school food environment policy interventions and dietary habits, adiposity, or metabolic risk factors in children (N = 91 studies).

Study	Design^a^	Country	Policy Type^b^	Policy Contribution^c^	Additional Intervention Components^d^	Intervention Level	Intervention Duration^e^	Quality Score^f^
**Amin 2015 ^[^^96^^]^**	QED, no C	US	SMS	High	None	Law, national	8	3
**Anderson 2005 ^[^^73^^]^**	RCT	UK	DP; SMS	Low	Edu; Mrk; Fml	Program, local	9	4
**Anderson 2013 ^[^^97^^]^**	QED, no C	US	SMS	Low	Edu	Law, national	50	3
**Ashfield-Watt 2009 ^[^^37^^]^^g^**	RCT	New Zealand	DP	High	None	Program, local	2.3	4
**Ask 2010 ^[^^98^^]^^h^**	RCT	Norway	SMS	High	None	Program, local	4	5
**Bae 2012 ^[^^76^^]^^h^**	QED, no C	South Korea	CFS	Medium	Edu; Lbl	Law, national	36	3
**Bartholomew 2006 ^[^^99^^]^**	RCT	US	SMS	Low	Edu; Fml	Program, local	12	3
**Bartlett, 2013 ^[^^38^^]^**	QED, C	US	DP	High	Edu; Mrk; Fml	Law, national	33	4
**Bauhoff 2013 ^[^^77^^]^**	QED, C	US	CFS	High	None	Policy, local	27	1
**Bere 2005 ^[^^41^^]^**	RCT	Norway	DP	Medium	Edu	Program, statewide	8	5
**Bere 2006 ^[^^40^^]^^g^**	RCT	Norway	DP	Medium	Edu	Program, statewide	8	5
**Bere 2007 ^[^^42^^]^^g^^,^^h^**	RCT	Norway	DP	Medium	Edu	Program, statewide	NA^i^	5
**Bere 2010 ^[^^10^^]^**	QED, C	Norway	DP	High	None	Policy, national	12	3
**Bere 2015 ^[^^39^^]^^g^^,^^h^**	RCT	Norway	DP	Medium	Edu	Program, statewide	NA^i^	5
**Bergman 2014 ^[^^100^^]^**	QED, no C	US	SMS	High	None	Law, national	8	3
**Blum 2008 ^[^^78^^]^**	QED, C	US	CFS	High	None	Program, local	9	1
**Bogart 2016 ^[^^43^^]^^h^**	RCT	US	DP	Low	Mrk; Fml; Bhv	Program, local	1.2	5
**Bonsergent 2013 ^[^^44^^]^**	RCT	France	DP	Low	Edu; Mrk; Fml	Program, local	33	5
**Burgess-Champoux 2008 ^[^^101^^]^**	QED, C	US	SMS	Low	Edu; Fml	Program, local	4	3
**Cohen 2012 ^[^^104^^]^**	QED, C	US	SMS	High	None	Program, local	21	4
**Cohen 2014 ^[^^102^^]^**	RCT	US	SMS	Low	Edu; Mrk; Fml	Program, local	9	5
**Cohen 2014 ^[^^103^^]^**	QED, no C	US	SMS	High	None	Law, national	NA^k^	3
**Coleman 2012 ^[^^66^^]^**	RCT	US	DP; CFS	Low	Edu; Mrk; Fml; Bhv; Env	Program, local	21	4
**Coyle 2009 ^[^^45^^]^**	QED, no C	US	DP	Medium	Edu; Mrk	Program, statewide	9	3
**Cradock 2011 ^[^^79^^]^**	QED, C	US	CFS	High	None	Policy, citywide	19	3
**Cullen 2008 ^[^^90^^]^**	QED, no C	US	CFS; SMS	High	None	Policy, statewide	45	3
**Cullen 2015 ^[^^105^^]^**	QED, C	US	SMS	High	None	Program, local	NA^k^	4
**Cummings 2014 ^[^^106^^]^**	QED, no C	US	SMS	High	Mrk	Program, local	12	3
**Davis 2009 ^[^^46^^]^^h^**	QED, C	US	DP	High	None	Policy, local	12	3
**Dwyer 1996 ^[^^107^^]^**	RCT	US	SMS	Low	Edu; Fml	Program, local	33	5
**Eagle 2013 ^[^^67^^]^**	QED, no C	US	DP; CFS	Low	Edu; Mrk; Bhv	Program, local	2.3	3
**Elbel 2015 ^[^^47^^]^**	QED, C	US	DP	High	None	Program, local	3	4
**Eriksen 2003 ^[^^48^^]^**	QED, C	Denmark	DP	High	None	Program, local	1	2
**Fiske 2004 ^[^^49^^]^^h^**	RCT	US	DP	Low	Fml; Env	Program, local	1	3
**Fogarty 2007 ^[^^50^^]^^g^**	RCT	UK	DP	High	None	Policy, national	12	3
**Folta 2013 ^[^^108^^]^**	QED	US	SMS	Low	Edu; Mrk; Fml; Lbl; Bhv; Env	Program, citywide	21	2
**Foster 2008 ^[^^80^^]^**	RCT	US	CFS	Low	Edu; Mrk; Fml	Program, local	21	5
**Foster 2010 ^[^^91^^]^**	RCT	US	CFS; SMS	Low	Edu; Mrk; Bhv	Policy, statewide	24	5
**French 2004 ^[^^51^^]^^h^**	RCT	US	DP	Medium	Mrk	Program, citywide	21	3
**Fung 2013 ^[^^74^^]^**	QED, no C	Canada	DP; CFS; SMS	Low	Edu; Mrk; Bhv	Policy, statewide	60	2
**van de Gaar 2014 ^[^^64^^]^**	RCT	Netherlands	DP	Low	Edu; Mrk; Fml	Program, local	9	5
**Haroun 2011 ^[^^109^^]^**	QED, no C	UK	SMS	High	None	Policy, statewide	7	2
**He 2009 ^[^^52^^]^**	RCT	Canada	DP	High	None	Program, local	12	4
**Hollar 2010 ^[^^110^^]^**	QED	US	SMS	Low	Edu; Mrk; Fml; Lbl; Bhv; Env	Program, local	21	2
**Hoppu 2010 ^[^^75^^]^**	RCT	Finland	DP; CFS; SMS	Low	Edu; Fml; Bhv	Program, local	9	4
**Jensen 2012 ^[^^81^^]^**	QED, no C	US	CFS	High	None	Policy, statewide	11	3
**Kaufman 2011 ^[^^93^^]^**	RCT	US	CFS; SMS	Low	Edu; Mrk; Bhv	Program, local	24	5
**Kim 2012 ^[^^68^^]^^h^**	QED, C	South Korea	DP; CFS	Low	Edu; Mrk; Lbl	Program, local	2.3	3
**Kocken 2012 ^[^^69^^]^^h^**	RCT	Netherlands	DP; CFS	High	None	Program, local	5	4
**Kocken 2015 ^[^^70^^]^^h^**	RCT	Netherlands	DP; CFS	High	None	Program, local	5	4
**Loughridge 2005 ^[^^53^^]^^h^**	QED, no C	UK	DP	High	None	Program, local	1	2
**Luepker 1996 ^[^^111^^]^**	RCT	US	SMS	Low	Edu; Fml	Program, local	33	5
**Lytle 2004 ^[^^54^^]^**	RCT	US	DP	Low	Edu; Fml; Bhv	Program, citywide	24	5
**Marcus 2009 ^[^^94^^]^**	RCT	Sweden	CFS; SMS	Low	Fml; Env	Program, local	47	4
**Mobley 2012 ^[^^92^^]^**	RCT	US	CFS; SMS	Low	Edu; Mrk; Fml; Bhv	Program, local	18	5
**Moore 2008 ^[^^71^^]^**	RCT	UK	DP; CFS	High	None	Program, local	9	5
**Muckelbauer 2009 ^[^^55^^]^**	RCT	Germany	DP	Medium	Edu	Program, local	10	2
**Mullally 2010 ^[^^95^^]^**	QED, no C	Canada	CFS; SMS	Low	Edu; Mrk; Ecn	Policy, statewide	9	2
**Murphy 2011 ^[^^112^^]^^h^**	RCT	UK	SMS	High	None	Program, statewide	12	4
**Nicklas 1996 ^[^^114^^]^**	RCT	US	SMS	Medium ^j^ Low ^j^	Edu Edu;Fml	Program, local	33	5
**Olsho 2015 ^[^^56^^]^^h^**	QED, C	US	DP	High	Edu; Mrk; Fml	Law, national	9	4
**Osganian 2003 ^[^^115^^]^^g^^,^^h^**	RCT	US	SMS	Low	Edu; Fml	Program, local	NA^i^	5
**Palakshappa 2016 ^[^^82^^]^**	QED, C	US	CFS	High	None	Law, statewide	18	3
**Perry 2004 ^[^^116^^]^**	RCT	US	SMS	Low	Mrk; Bhv	Program, local	21	4
**Rahmani 2011 ^[^^57^^]^^h^**	RCT	Iran	DP	High	Edu; Mrk	Program, local	3	3
**Ransley 2007 ^[^^58^^]^**	QED, C	UK	DP	High	Edu; Mrk; Fml	Program, local	9	3
**Reinaerts 2008 ^[^^59^^]^^g^**	QED, C	Netherlands	DP	Medium	Edu; Mrk; Fml	Program, statewide	8	3
**Sanchez-Vaznaugh 2010 ^[^^83^^]^**	QED, no C	US	CFS	High	None	Policy, statewide	46	3
**Sanchez-Vaznaugh 2015 ^[^^84^^]^**	QED, no C	US	CFS	High	None	Policy, statewide	46	3
**School Food Trust 2011 ^[^^113^^]^**	QED, no C	UK	SMS	High	None	Law, national	19	4
**Schwartz 2009 ^[^^85^^]^^h^**	QED, C	US	CFS	High	None	Program, local	12	4
**Schwartz 2015 ^[^^117^^]^^h^**	QED, C	US	SMS	High	None	Law, national	20	3
**Schwartz 2016 ^[^^60^^]^^h^**	QED, no C	US	DP	High	None	Program, local	NA^k^	4
**Simons-Morton 1991 ^[^^118^^]^**	QED, C	US	SMS	Medium	Edu	Program, local	21	2
**Slusser 2007 ^[^^61^^]^**	QED, no C	US	DP	High	Edu; Mrk; Bhv; Env	Program, local	9	2
**Snyder 1992 ^[^^119^^]^**	QED, no C	US	SMS	High	Edu	Program, local	4	2
**Spence 2013 ^[^^120^^]^**	QED, no C	UK	SMS	High	None	Law, national	9	3
**Spence 2014 ^[^^122^^]^**	QED, no C	UK	SMS	High	None	Law, national	9	4
**Spence 2014 ^[^^121^^]^**	QED, no C	UK	SMS	High	None	Law, national	NA^k^	4
**Story 2003 ^[^^123^^]^**	RCT	US	SMS	Medium	Edu; Fml	Program, local	33	5
**Taber 2012 ^[^^86^^]^**	QED, C	US	CFS	High	None	Law, statewide	40	3
**Taber 2012 ^[^^87^^]^**	QED, C	US	CFS	High	None	Law, statewide	9	3
**Tak 2009 ^[^^62^^]^**	QED, C	Netherlands	DP	Medium	Edu	Program, local	21	2
**te Velde 2008 ^[^^63^^]^**	RCT	Netherlands	DP	Low	Edu; Mrk; Fml	Program, local	21	4
**Visscher 2010 ^[^^65^^]^^h^**	QED, no C	Netherlands	DP	High	None	Program, local	3	1
**Whitaker 1993 ^[^^124^^]^**	QED, no C	US	SMS	High	None	Program, local	8	3
**Williams 2002 ^[^^125^^]^**	QED, C	US	SMS	High	Edu	Program, local	21	3
**Williamson 2007 ^[^^126^^]^**	RCT	US	SMS	Low	Mrk; Fml	Program, local	21	4
**Williamson 2012 ^[^^88^^]^**	RCT	US	CFS	High ^j^ Low ^j^	None Edu; Fml	Policy, statewide	33	3
**Woodward-Lopez 2010 ^[^^89^^]^^h^**	QED, no C	US	CFS	High	None	Law, statewide	9	3
**Wordell 2012 ^[^^72^^]^^h^**	QED, C	US	DP; CFS	High	None	Program, local	33	4

^a^ We included all interventional studies including randomized controlled trials (RCTs) or quasi-experimental designs with (QED) or without an external control group (QED, no C) that assessed the impact of school food environment policy on dietary intake, adiposity, or metabolic outcomes in children. Specific interventions were represented by more than 1 study if different outcomes (e.g., intake vs content, school vs habitual) were reported.

^b^ School food environment policy interventions included the direct provision of healthful foods and beverages (DP), competitive food and beverage standards (CFS), and/or school meal standards (SMS).

^**c**^ Multi-component strategies were included only if the food environment policy was a major component, judged qualitatively to be at least 30% of the overall intervention. The relative contribution of the food environment policy component to the overall intervention was qualitatively assessed by each reviewer, independently and in duplicate, based on the number, types, and intensity of additional intervention components, as low (30 to <60%), medium (60 to <90%), and high (≥90%). Single-component strategies received 100%.

^**d**^ Additional intervention components in multi-component strategies included education (nutrition curricula) (Edu), promotion/ marketing (Mrk), family/ parent outreach (Fml), point-of-purchase labeling (Lbl), behavioral techniques (Bhv), other environmental change (Env), and economic incentive (Ecn).

^**e**^ Intervention duration (in months) was estimated from the end of data collection and start date of the intervention as reported. Periods that schools are closed (e.g., summer, holidays) were not taken into account in such estimations.

^f^ Quality assessment was performed by review of study design, assessment of exposure, assessment of outcome, control of confounding, and evidence of bias. Each of the 5 quality criteria was evaluated and scored on an integer scale (0 or 1, with 1 being better) and summed; quality scores from 0 to 3 were considered lower quality and 4 to 5 higher quality.

^g^ Additionally or exclusively [39,115] reported sustainability effects (i.e., change in reported outcome after the end of the intervention). Of these, 3 studies [37,50,59] within the same strategy (DP) could be meta-analyzed for changes in total fruit intake. One study, which published findings separately 1 yr, 3 yrs and 7 yrs after the intervention was not included in pooled analyses, as the reported outcome was fruit and vegetable intake combined [39,40,42]; and one study reported only sustainability effects within the SMS strategy [115].

^h^ Studies only included in qualitative assessment.

^i^ Reported only sustainability effects 36 months[42], 84 months [39] and 60 months [115] after the program was terminated; not included in pooled analyses.

^j^ Two intervention arms with overlapping components were available. We included the intervention arm with greatest relative contribution of food environment policy to the overall intervention.

^k^ Data collection period was not clearly defined.

### Direct provision of healthful foods and beverages

Interventions providing healthful foods/beverages were mainly in classrooms (“direct” provision) or via increased availability in cafeterias, tuck shops or vending machines (“indirect” provision) (Table 1). F&V were most common.

#### Fruits

**Fig 2:**
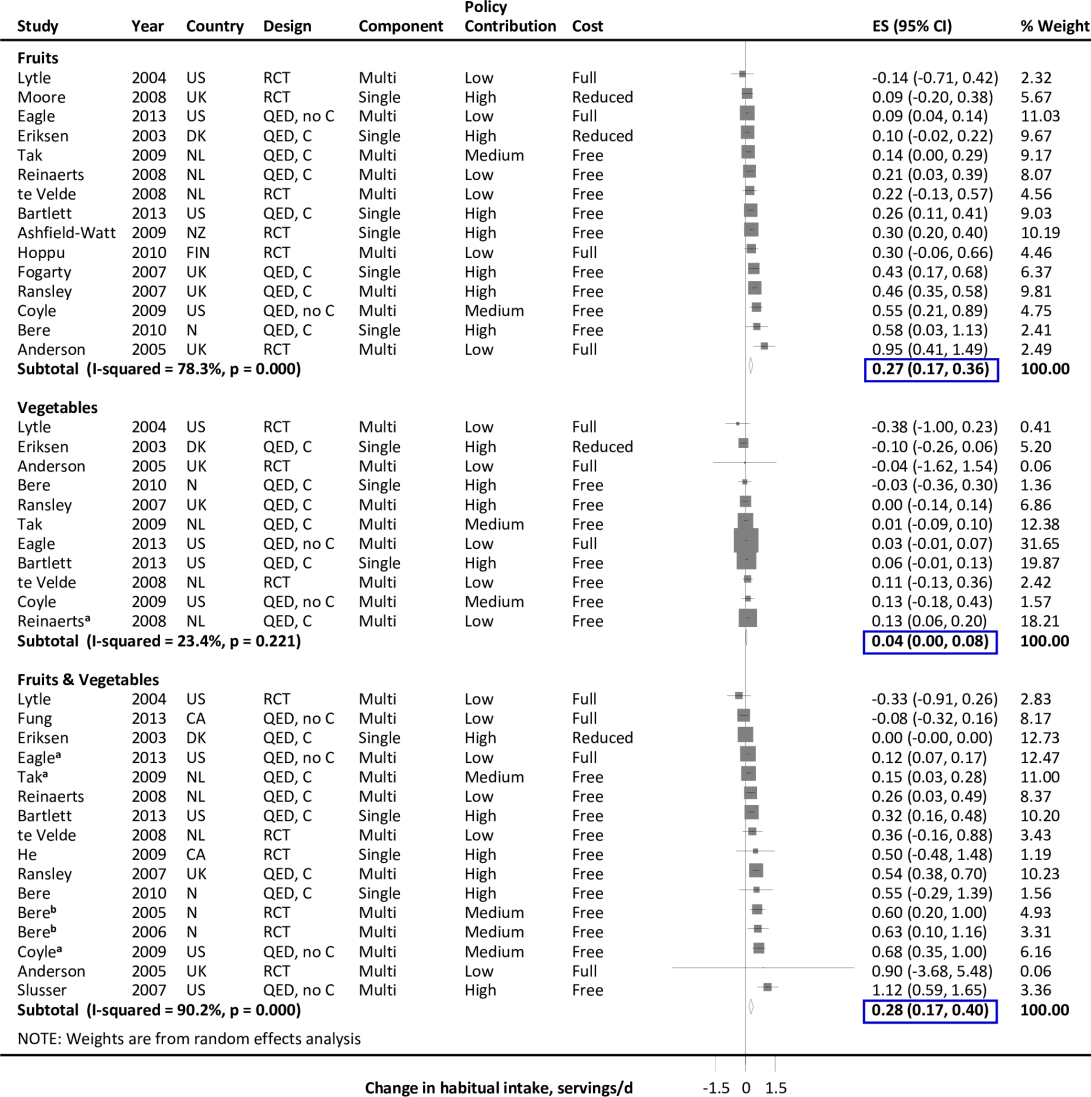
Effect of direct provision of fruits and vegetables in schools on fruit and vegetable intake in children. Intakes represent habitual (not just in-school) consumption. Solid squares represent study specific continuous changes in reported intakes; and lines, 95% confidence intervals (Cis). Vertical line represents pooled effect size (ES); and open diamond, corresponding 95% CI. Multi-component strategies were included only if the food environment policy was a major component, judged qualitatively to be at least 30% of the overall intervention. The relative contribution of the food environment policy component to the overall intervention was qualitatively assessed as low (30 to <60%), medium (60 to <90%), and high (≥90%). ^a^ A single estimate was obtained by summing separately reported outcomes (n = 2) that their total aligned to the single optimal definition (i.e., total vegetables, combined fruits and vegetables). ^b^ Same intervention reporting outcomes for different counties and ages. RCT, randomized controlled trial; QED, quasi-experimental intervention with external control group; QED, no C, quasi-experimental intervention without external control group; CA, Canada; DK, Denmark; F, Finland; N, Norway; NL, Netherlands; NZ, New Zealand; UK, United Kingdom; US, United States of America.

Pooling 6 randomized and 9 quasi-experimental interventions with average duration 12 months, habitual fruit intake increased by 0.27 servings/d (95%CI: 0.17, 0.36) (Fig 2, Table B in S1 File). Effects were similar in randomized vs. quasi-experimental studies (Table 2, Figure A in S1 File). Effects appeared potentially higher with free provision [10,37,38,45,50,58,59,62,63] vs. reduced [48,71] or full [54,67,73,75] price, but this heterogeneity was not statistically significant (P = 0.07) (Figure A in S1 File). Findings were also similar in direct provision only vs. multi-component interventions; or in “direct” (n = 10; 0.29 (0.19, 0.39)) [10,37,38,45,48,50,58,59,62,63] vs. “indirect” (n = 5; 0.21 (-0.02, 0.44)) [54,67,71,73,75] interventions. Results were similar in 5 studies [10,37,38,45,71] assessing in-school fruit consumption (Table B in S1 File). Three studies [37,50,59] assessed sustainability at 6 weeks [37] or 12 months [50,59] after direct provision was removed; no significant effect was seen (-0.18 (-0.51, 0.15)).

**Table 2:** Prespecified sources of heterogeneity explored among interventions evaluating the effect of direct provision of fruits and vegetables in schools on habitual fruit and vegetable intake in children.

Heterogeneity sources ^a^	Fruits, servings (80 g)/d		Vegetables, servings (80 g)/d		Combined fruits & vegetables, servings (80 g)/d	
	N (n) ^b^	Mean (95% CI) ^c^	N (n)	Mean (95% CI) ^c^	N (n)	Mean (95% CI) ^c^
Overall	15 (15)	0.27 (0.17, 0.36)	11 (11)	0.04 (0.01, 0.08)	16 (16)	0.28 (0.17, 0.40)
Study design						
**RCT**	6 (6)	0.27 (0.09, 0.45)	3 (3)	0.02 (-0.25, 0.29)	6 (6)	0.37 (0.05, 0.69)
**QED**	9 (9)	0.27 (0.15, 0.39)	8 (8)	0.04 (0.00, 0.09)	10 (10)	0.26 (0.14, 0.38)
Region						
**US/Canada**	4 (4)	0.21 (0.02, 0.40)	4 (4)	0.04 (0.00, 0.07)	7 (7)	0.29 (0.07, 0.51)
**Europe/New Zealand**	11 (11)	0.29 (0.18, 0.39)	7 (7)	0.04 (-0.04, 0.11)	9 (9)	0.33 (0.13, 0.53)
Type of intervention ^d^						
**Food policy only**	5 (5)	0.25 (0.10, 0.39)	2 (2)	-0.09 (-0.23, 0.06)	3 (3)	0.03 (-0.12, 0.18)
**Multi-component**	10 (10)	0.28 (0.14, 0.41)	9 (9)	0.05 (0.02, 0.09)	13 (13)	0.33 (0.19, 0.47)
Non-dietary targets ^e^						
**No**	14 (14)	0.29 (0.19, 0.38)	10 (10)	0.05 (-0.01, 0.10)	14 (14)	0.33 (0.16, 0.50)
**Yes**	1 (1)	n/a	1 (1)	n/a	2 (2)	0.12 (0.08, 0.17)
No of environmental strategies ^f^						
**1**	11 (11)	0.28 (0.18, 0.38)	9 (9)	0.05 (-0.01, 0.10)	13 (13)	0.38 (0.20, 0.56)
**>1**	4 (4)	0.26 (0.00, 0.52)	2 (2)	0.03 (-0.01, 0.07)	3 (3)	0.07 (-0.06, 0.21)
School level ^g^						
**Primary**	10 (10)	0.24 (0.15, 0.34)	7 (7)	0.05 (-0.01, 0.11)	12 (12)	0.29 (0.13, 0.45)
**Secondary**	3 (3)	0.09 (0.04, 0.14)	2 (2)	-0.06 (-0.39, 0.27)	2 (2)	-0.002 (-0.39, 0.39)
**Preschool & primary**	1 (1)	n/a	1 (1)	n/a	1 (1)	n/a
**Primary & secondary**	1 (1)	n/a	1 (1)	n/a	1 (1)	n/a
Quality score ^h^						
**Low**	8 (8)	0.27 (0.14, 0.41)	7 (7)	0.04 (-0.02, 0.09)	10 (10)	0.26 (0.13, 0.38)
**High**	7 (7)	0.27 (0.14, 0.39)	4 (4)	0.06 (-0.01, 0.12)	6 (6)	0.36 (0.10, 0.61)
Cost of provision ^i^						
**Free**	9 (9)	0.32 (0.22, 0.41)	7 (7)	0.07 (0.03, 0.11)	10 (10)	0.41 (0.26, 0.55)
**Reduced/ Full**	6 (6)	0.15 (0.02, 0.27)	4 (4)	-0.01 (-0.12, 0.09)	6 (6)	0.07 (-0.05, 0.20)

^a^ Results are presented for selected heterogeneity sources (common across the three strategies of school food environment policies identified -Tables C and D in S1 File–with the exception of “Cost of provision”, specific to this strategy only) for the outcomes with the largest numbers of estimates. For all other outcomes not presented, no significant heterogeneity sources were identified. None of the identified differences by subgroups were statistically significant by meta-regression (P-heterogeneity>0.05 each).

^b^ Number of estimates (n, values in parentheses) can be higher than number of studies (N) included in the meta-analyses if multiple intervention groups or multiple comparisons were available from the same study

^c^ Study-specific effect sizes were pooled using stratified inverse-variance weighted random-effect models (metan command in STATA). Effect sizes correspond to mean changes standardized across studies to consistent units; and precision estimates to 95% confidence intervals (CIs).

^d^ Single-component interventions consisted only of the school food environment policy. Multi-component interventions were included only if the food environment policy was a major component, judged qualitatively to be at least 30% of the overall intervention. Additional potential components included education, food/menu labeling, etc. (see Table 1).

^e^ In addition to the dietary targets, specific interventions also targeted non-dietary targets, such as physical activity and smoking.

^f^ School food environment policy strategies included direct provision of healthful foods, quality standards for competitive foods/ beverages, and quality standards for school meals.

^g^ Preschool: 2–4 years old; primary: 5–11 years old; secondary level: 12–18 years old.

^h^ Quality assessment was performed by review of study design, assessment of exposure, assessment of outcome, control of confounding, and evidence of bias. Each of the 5 quality criteria was evaluated and scored on an integer scale (0 or 1, with 1 being better) and summed; quality scores from 0 to 3 were considered lower quality and 4 to 5 higher quality.

^i^ Provision of fruits and vegetables could be either free (mainly when the intervention included direct provision of fruits and vegetables in the classroom) or it could come at reduced/full price (mainly when the intervention included indirect provision through increasing the availability of fruits and vegetables in cafeterias, tuck shops or vending machines).

CI, Confidence Intervals; RCT, randomized controlled trial; QED, quasi-experimental intervention.

#### Vegetables

Pooling 3 randomized and 8 quasi-experimental interventions with average duration 13.4 months, habitual vegetable intake was slightly increased (0.04 servings/d (0.01, 0.08)) (Fig 2). In 7 interventions providing free vegetables, effects appeared higher, although this heterogeneity was not statistically significant (P = 0.22) (Table 2, Figure B in S1 File). Findings were similar stratified by other study characteristics and in 3 studies [10,38,45] assessing in-school intake (Table B in S1 File).

#### Combined fruits and vegetables

Sixteen studies (6 randomized, 10 quasi-experimental) assessed combined F&V intake, with average duration 15.4 months (11 of these studies also separately evaluated fruits or vegetables, above). Combined intake increased by 0.28 servings/d (n = 16 (0.17, 0.40)) (Fig 2). Findings were not significantly different in randomized vs. quasi-experimental studies or by other population or intervention characteristics (Table 2, Figure C in S1 File). In 6 studies assessing in-school consumption [10,38,40,41,45,52], combined F&V intake increased by 0.38 servings/d (n = 6 (0.23, 0.53)) (Table B in S1 File).

#### Total calories

Habitual caloric intake was reported in 6 studies [38,58,61,73–75], yet wasn’t a target of direct provision in any of these. Pooling studies, no significant effect on habitual caloric intake was identified (-56 kcal/d; -174, 62) (Table B in S1 File). Only 1 study reported school caloric intake [56], which was unchanged.

#### Water

Five studies increased access to free water mainly through installment of water coolers [47,53,55,64,65]. Of these, 3 reported nonsignificant trends toward increased habitual water consumption (0.33 glasses/d (-0.27, 0.93)) [47,55,64] (Table B in S1 File); and 3 reported changes in uptake, which decreased in 2 studies [55,65] and increased in one [53].

#### Adiposity and metabolic measures

Four studies combining provision of fruits and vegetables with additional competitive food/beverage standards evaluated overweight or obesity, with average duration 26.8 months (range 2.3 to 60) [44,66,67,74]. Improvements were not identified in odds of overweight/obesity (n = 2; 1.04 (0.91, 1.19)) [44,66], overweight (n = 1; 1.03 (0.94, 1.12)) [74], or obesity (n = 2; 1.25 (1.07, 1.46)) [66,74]; BMI (n = 3; 0.19 kg/m^2^ (-0.12, 0.50)) [44,66,67]; or BMI z-score (n = 2; 0.01 (-0.04, 0.05)) [44,66]. Another 3 studies [43,55,60] focusing on water provision reported improvements in BMI z-score [60], prevalence of overweight/obesity [60] and odds of overweight [55], while obesity prevalence [60] and BMI percentile were unchanged [43]. Only 1 study [67] evaluated metabolic risk factors, finding significant decreases in total cholesterol, LDL cholesterol, and triglycerides, and blood pressure.

#### Other endpoints

Three studies [46,68,72] evaluated odds of consuming F&V [72] or varying percentage changes in F&V intakes, reported dichotomously [46,68]; these outcomes were generally not significantly improved. One study reported only sustainability data for F&V intake after end-intervention, finding sustained benefits for both in-school intake at 3 years [42] and habitual F&V intake at 3 [42] and 7 years [39] although this weakened over time. A few interventions provided low-fat/low-calorie items [49,51,69], or milk [57]. No significant improvements were found in consumption of low-fat items. A milk provision study in Iran aimed to increase students’ weight, which was achieved.

### Competitive food and beverage standards

Competitive food/beverage policies generally targeted SSBs and unhealthy snacks (Table 1). Strategies included product-specific restrictions; standards on nutrients, calories, or portion sizes; or both. All were performed prior to implementation of US national Smart Snacks guidelines in 2014.

#### Sugar-sweetened beverages

**Fig 3:**
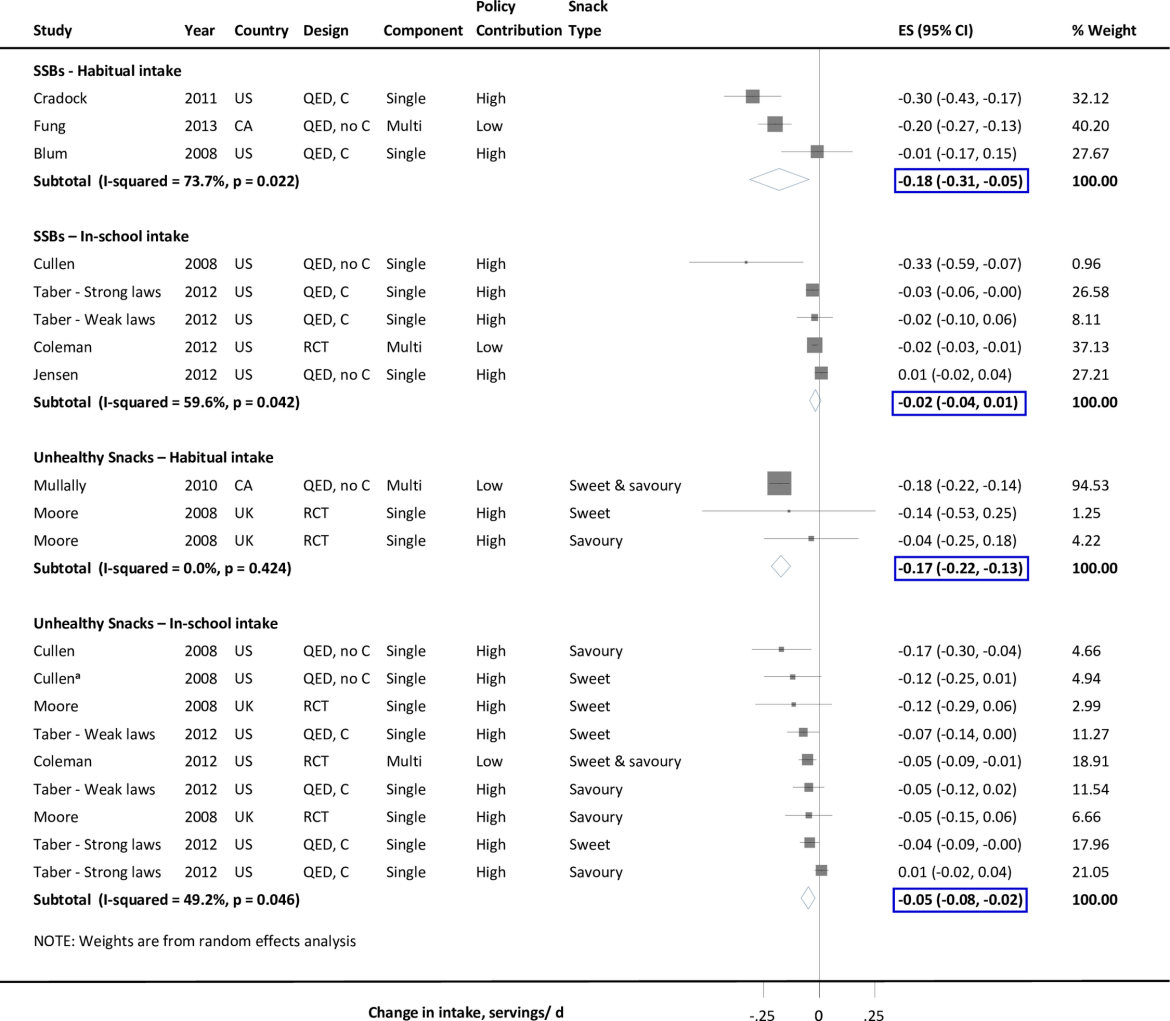
Effect of competitive food and beverage standards in schools on sugar-sweetened beverage and unhealthy snack intake in children. Intakes represent habitual or total in-school consumption, except for 1 study that assessed in-school lunch intake. Solid squares represent study specific continuous changes in reported intakes; and lines, 95% confidence intervals (Cis). Vertical line represents pooled effect size (ES); and open diamond, corresponding 95% CI. Multi-component strategies were included only if food environment policy was a major component, judged qualitatively to be at least 30% of the overall intervention. The relative contribution of the food environment policy component to the overall intervention was qualitatively assessed as low (30 to <60%), medium (60 to <90%), and high (≥90%). ^a^ A single estimate was obtained by summing separately reported outcomes (n = 2) that their total aligned to the single optimal definition (i.e., sweet snacks). SSBs, sugar-sweetened beverages; RCT, randomized controlled trial; QED, quasi-experimental intervention with external control group; QED, no C, quasi-experimental intervention without external control group; CA, Canada; UK, United Kingdom; US, United States of America.

Three interventions found decreased habitual SSB intake of 0.18 servings/d (n = 3 (-0.31, -0.05)) (Fig 3). In contrast, 4 separate studies assessing in-school intake did not identify a significant effect (n = 5; -0.02 servings/d (-0.04, 0.01)). No significant heterogeneity sources were identified (Table C and Figure D in S1 File).

#### Unhealthy snacks

Two interventions assessed habitual intake, which decreased by 0.17 servings/d (n = 3 (-0.22, -0.13)) (Fig 3). Four studies with 5 separate intervention arms assessed in-school intake, which decreased by 0.05 servings/d (n = 9; -0.08, -0.02) (Fig 3). No significant heterogeneity sources were identified (Table C and Figure D in S1 File).

#### Total calories

Habitual caloric intake was reported in 5 studies [74,75,80,86,93], with no significant effect (-79 kcal/d; -179, 21) (Table B in S1 File). Findings were not significantly different in 2 studies [86,93] that specifically targeted calories (-40 kcal/d; -185, 104) or in 3 (2 additional) studies that assessed in-school lunch caloric intake [86,88,90].

#### Other targeted dietary factors

Other targeted diet factors included total fat [88,90] and saturated fat [86,88] intake; habitual and in-school lunch total fat intake decreased (n = 3), but not in-school lunch saturated fat intake (n = 2) (Table B in S1 File).

#### Adiposity and metabolic measures

Several studies assessed the prevalence or odds ratios of childhood overweight (n = 6 and n = 6 estimates, respectively), obesity (n = 10, n = 8), or overweight/obesity (n = 5, n = 2) (Figures G and H in S1 File), as well as BMI (n = 6; Figure I in S1 File) and BMI z-score (n = 5; Figure J in S1 File). Durations ranged from 2.3 to 69 months (mean 31.5). Competitive food/beverage standards did not significantly reduce any of these measures (Table B in S1 File), although the central effect estimate often tended to be slightly and nonsignificantly lower. Prevalence of overweight/obesity was nonsignificantly higher across 5 studies evaluating this outcome (n = 5; 0.24%; -0.54, 1.02), largely driven (70.51% of the weighted estimate) by 1 quasi-experimental study [84] that compared changes in rates among schoolchildren in California (n = ~600,000). Only 2 studies evaluated effects on metabolic risk factors and could not be pooled [67,91]; individually, these found significant improvements in various risk factors assessed [67] other than fasting glucose [91].

#### Other endpoints

Eight studies [68–70,72,76,85,89,92] reported odds of consuming SSBs and unhealthy snacks [72], changes in total caloric and total fat meal content [92], or changes in SSBs and unhealthy snack intakes reported dichotomously (e.g., percentage of sales, prevalence of students, score expressing frequency of intake) [68–70,76,85,89] that could not be meta-analyzed due to outcome heterogeneity. Qualitatively, these studies reported conflicting findings regarding SSB and unhealthy snack intake, with some reporting decreases [76,85,89], others showing no change [68–70,72], and one showing unhealthy snack increases [68]; total caloric and total fat school meal content decreased.

### School meal standards

Policies on school meal (mainly lunch) standards (foods, nutrients/energy) generally targeted F&V, dietary fats, and sodium (Table 1). Five studies evaluated implementation of the 2012 US school lunch guidelines, while all studies were performed prior to the implementation of the revised 2015 UK school meal standards.

#### Fruits and vegetables

Standards on F&V (e.g., serve at least one fruit or vegetable daily) generally targeted lunch, either alone or combined with direct provision. Habitual fruit intake increased by 0.76 servings/d (n = 2 (0.37, 1.16)) [73,102]; with nonsignificant trends toward increased habitual vegetable (n = 2; 0.30 servings/d (-0.001, 0.59)) [73,102] and F&V (n = 5; 0.12 servings/d (-0.08, 0.31)) consumption (Table B in S1 File) [73,74,95,102,108]. Findings were similar restricting to 3 studies [95,102,108] that did not include direct provision (n = 3; 0.23 servings/d of F&V; (-0.06, 0.51)). In one study assessing prevalence of students selecting F&V in lunch [117], fruit selection increased, while vegetable selection decreased.

#### Dietary fats

**Fig 4:**
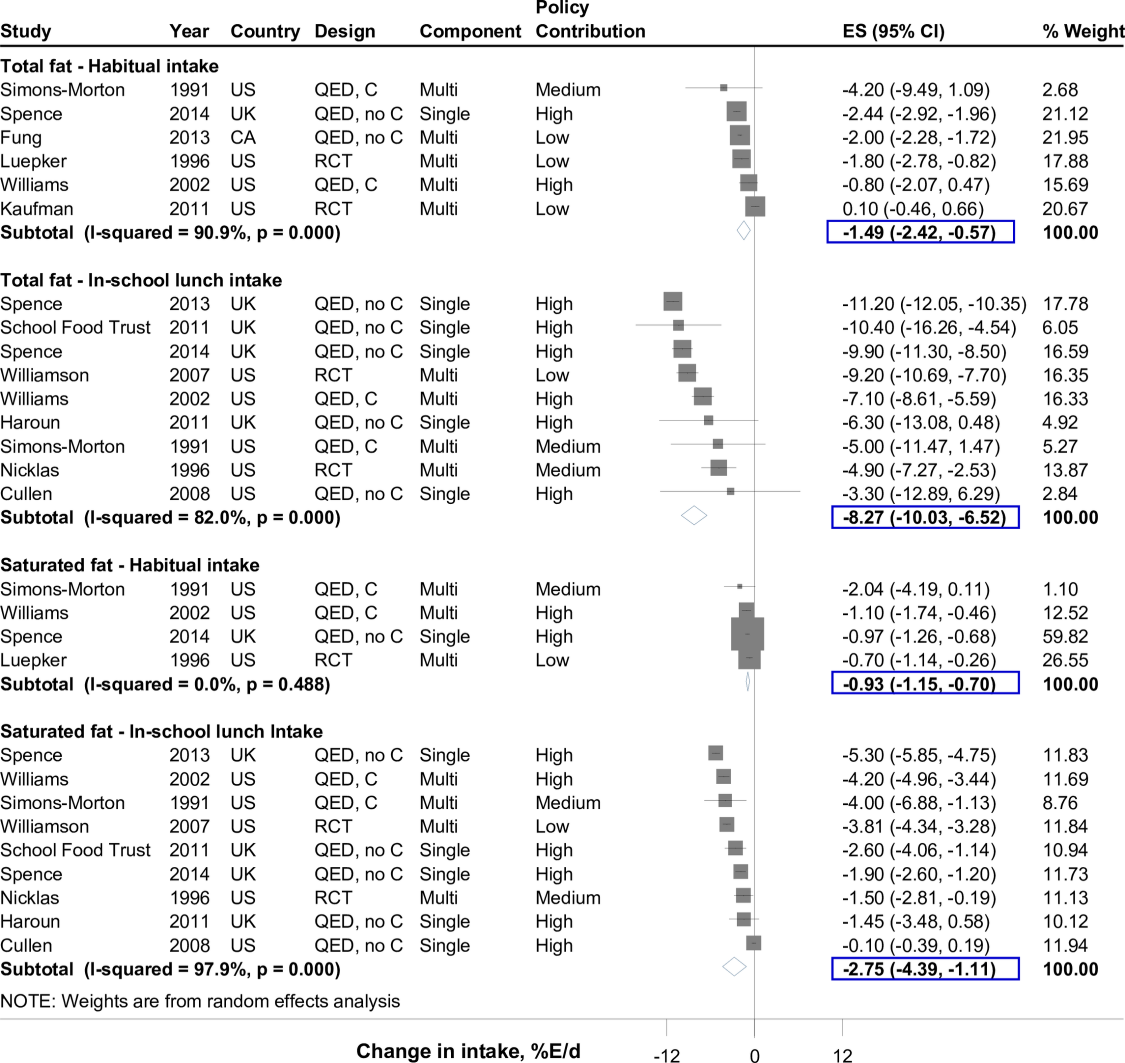
Effect of school meal standards on total fat and saturated fat intake in children. Intakes represent habitual or in-school lunch consumption. Solid squares represent study specific continuous changes in reported intakes; and lines, 95% confidence intervals (Cis). Vertical line represents pooled effect size (ES); and open diamond, corresponding 95% CI. Multi-component strategies were included only if the food environment policy was a major component, judged qualitatively to be at least 30% of the overall intervention. The relative contribution of the food environment policy component to the overall intervention was qualitatively assessed as low (30 to <60%), medium (60 to <90%), and high (≥90%). In secondary analysis, in-school meal (lunch or breakfast) consumption decreased for total fat by 7.12% energy (%E)/d (N = 10; -9.48, -4.75) and for saturated fat by 2.46%E/d (N = 10; -4.04, -0.89). RCT, randomized controlled trial; QED, quasi-experimental intervention with external control group; QED, no C, quasi-experimental intervention without external control group; CA, Canada; UK, United Kingdom; US, United States of America.

Most studies specified target levels for dietary fats, which were generally consistent across studies; these ranged from 30–35%E/lunch for total fat and 10–11%E/lunch for saturated fat. Six studies assessed habitual total fat, which decreased by 1.49%E (-2.42, -0.57) (Fig 4). In g/d, the reduction in habitual fat intake was greater (~6 g/d total fat) in magnitude to achieved reductions in in-school meal content and intake (~3–4 g/d total fat) (Figure K in S1 File). Standards also reduced habitual saturated fat (n = 4; -0.93%E (-1.15, -0.70)), in-school lunch saturated fat (n = 9; -2.75%E (-4.39, -1.11)), and in-school meal (lunch or breakfast) saturated fat (n = 10; -2.46%E (-4.04, -0.89)) (Table B in S1 File). Again, absolute magnitudes of reduction were marginally higher for habitual intake (~3 g/d) compared with in-school meal intake and content (~1–2 g/d) (Figure L in S1 File). No significant heterogeneity sources were identified, with the exception of study region for in-school total fat intake (P = 0.042); larger reduction was observed for studies in Europe/New Zealand compared to US/Canada (Table D and Figures E and F in S1 File).

#### Total calories

School meal standards did not significantly decrease habitual caloric intake (n = 8; -38 kcal/d (-137, 62), in-school (lunch) calories (n = 11; -28 kcal/d (-76, 20)), or in-school (lunch+breakfast) calories (n = 12; -29 kcal/d (-76, 18)) (Figure M and Table B in S1 File). Results were similar in interventions specifically targeting total calories by aiming to provide adequate amounts of energy (habitual: n = 4; -19 kcal/d (-134, 95); in-school: n = 5; -60 kcal/d (-170, 50)). The magnitude of reduction was larger and significant for in-school meal content than for in-school meal intake or habitual intake (Figure M in S1 File). Differences were seen by study quality score for in-school lunch caloric intake (P = 0.01) but not for habitual caloric intake; nor for other heterogeneity sources (Table D in S1 File).

#### Sodium intake

Target levels for sodium content in school meals varied across studies, ranging from 200–1200 mg/meal. School meal standards for sodium decreased habitual intake (n = 4; -170 mg/d (-242, -98)), in-school lunch intake (n = 6; -227 mg/d (-384, -69)), and in-school meal (lunch+breakfast) intake (n = 7; -221 mg/d (-371, -71)) (Figure N and Table B in S1 File). The magnitude of reduction in sodium was similar for in-school meal content (Figure N in S1 File). No significant sources of heterogeneity were identified (Table D in S1 File).

#### Other targeted dietary factors

A few interventions set meal standards for other targets such as milk (one serving/d of milk/milk products; n = 2) [74,95], dietary fiber (grain-based foods with ≥2 g/serving of fiber; n = 7) [74,92,93,104,120–122], whole grains (increase whole grains, e.g., by 1 daily serving; n = 5) [74,92,101,102,104], or total carbohydrates (> = 50% of food energy; n = 3) [74,109,120]. These studies found increased habitual consumption of milk/milk products (0.22 cups/d; 0.17, 0.28) and in-school lunch consumption of carbohydrate (8.17%E/d; 0.70, 15.65), but not habitual or lunch intakes of dietary fiber (0.08 g/d (-0.84, 1.00); 0.55 (-1.90, 3.00); respectively) or habitual or lunch intakes of whole grains (0.14 servings/d (-0.11, 0.39); 0.49 (-0.37, 1.35); respectively). Three studies (n = 5 estimates) targeted the proportion of schoolchildren selecting “less healthy” options (e.g., desserts, high-fat entrees, starchy foods in oil) [99,109,113]; no significant effects were seen (Table B in S1 File).

#### Adiposity and metabolic measures

Six studies evaluated effects of school meal standards on adiposity, with average duration 34.3 months (range 4 to 60) [74,91,94,97,98,110]. Three of these [74,91,94] also combined competitive food/beverage standards. Two studies [97,110] assessed changes in BMI percentile, which decreased (-1.01, -1.62, -0.39), while other adiposity measures evaluated were unchanged (Table B in S1 File).

#### Other endpoints

Two studies reported dichotomous changes in healthy food (e.g., fruit, vegetable, bread, milk, cereal [98,112], reported as % meeting a threshold) that could not be meta-analyzed due to varying cutpoints. Results were conflicting, with a reduced overall healthy food score and a higher intake of healthy items at breakfast [112]. One study [115] reported only sustainability data, evaluating total calories, total fat, and saturated fat in lunches 5 years after school meal standards were removed, finding further decreases in %E from total and saturated fat, but increased caloric content.

### Publication bias

Visual inspection of funnel plots provided little evidence for publication bias (Figures O-Q in S1 File). Begg’s or Eggers test did not identify statistical evidence for publication bias.

## Discussion

This systematic review and meta-analysis is the first, to our knowledge, to determine quantitative effects of school food environment policies on children’s habitual dietary intakes in interventional studies. Direct provision policies increased fruit intake by 0.27 servings/d and vegetable intake by 0.04 servings/d, but not water intake. Competitive food/beverage standards reduced SSBs by 0.18 servings/d and unhealthy snacks by 0.17 servings/d. School meal standards increased fruit intake by 0.76 servings/d, reduced total fat intake by ~1.5% energy and saturated fat intake by ~1% energy, and reduced sodium by 170 mg/d. All of these policies influenced dietary composition, without altering total calories. Measures of adiposity were generally unchanged; and few studies assessed metabolic factors, with mixed findings.

We separately evaluated in-school vs. habitual intakes to determine effects on children’s overall nutritional habits, given potential for compensatory changes outside of school. For example, restricting SSBs or unhealthy snacks at school could lead to increased consumption after school or at home. Such compensation is suggested in some cases; for instance, school meal standards significantly reduced meal calorie content, but not in-school meal calorie intake or habitual calorie intake. Conversely, reductions were similar for in-school vs. habitual sodium intake, suggesting that sodium reduction at school does not lead to meaningful compensation elsewhere. For some policy outcomes, e.g. for competitive food standards and SSBs and snacks, the pooled findings from interventions evaluating in-school effects were smaller than those evaluating habitual intakes. These were generally different studies, suggesting possibly other differences in the types of studies evaluating in-school intakes. Overall, our results support the importance of schools as a setting to improve overall dietary habits of children within and outside school.

Our findings suggest efficacy of a range of food environment policies, including direct provision, competitive food/beverage standards, and school meal standards. The results for both direct provision and school meal standards suggest greater efficacy for fruit intake, compared with vegetables; consistent with greater palatability of many fruits and generally less need for preparation or cooking. Water intake was unchanged in the limited studies that assessed this outcome, likely further due to difficulties in assessing fluid intake and measurement error. Our findings further highlight key gaps for many other dietary targets, such as other healthier foods (e.g., legumes, whole grains, fish, yogurt) or less healthy foods (e.g., processed meats) or other nutrients of concern (e.g., calcium, vitamin D, potassium, unsaturated fats, fiber). Given updated Dietary Guidelines for Americans that focus on healthier foods, overall diet patterns, and specific nutrients of concern [127], future studies are needed to assess how school food environment policies impact these priorities.

Evidence on the health impact of policies targeting the school food environment is especially relevant and timely given the potentially evolving priorities of the new federal US administration. Congress did not reauthorize the Healthy, Hunger-Free Kids Act (HHFKA) as scheduled in Sept 2015, so the future of Smart Snack Standards, now covering 99% of public and 83% of private schools [12], remains uncertain. Further, current policy debates include a focus on weakening or eliminating national school lunch standards [15,16]. A recent analysis indicated that in-school selections have improved with the new lunch standards [128]. Our findings build upon and expand this prior work by demonstrating changes in actual habitual intake, further supporting efficacy of meal and snack standards and informing ongoing debates. Similarly, the current national FFVP only applies to elementary schools with high proportions of low-income students [9], about 4 million students across the US [129]. Our investigation supports efficacy of such direct provision programs, which should be considered for a broader range of elementary, middle, and high schools. Finally, while identified dietary improvements were meaningful at a population level, these will not fully address the suboptimal diets of most children. Thus, our results confirm a need for multiple programmatic and policy interventions, including within and outside schools, to improve children’s diets.

While several dietary benefits were confirmed, changes in adiposity metrics were generally not significant. This may be because such policies improve dietary quality or composition (more relevant for general and metabolic health) but not dietary quantity (more relevant for obesity, at least in the short- to intermediate-term). Because dietary composition influences numerous pathways for health and well-being, the absence of a documented effect on obesity does not preclude efficacy of these interventions. Few studies evaluated metabolic risk factors, for which improvements may be more readily detected compared with adiposity. Also, establishing lifelong healthier dietary habits may have benefits decades later, during adulthood. Our findings provide quantitative summaries of how school food environment policies affect specific dietary targets, allowing modeling of potential effects on childhood obesity and future diets and disease risk in adulthood.

Prior reviews of a more varied range of school interventions identified effects of similar magnitude for total F&V consumption [18,19,21]. A previous systematic review on competitive foods/beverages was qualitative, and included mostly cross-sectional studies in the US alone [24]. Similarly, another systematic review on school food environment was also qualitative, excluded direct provision studies, and grouped together various heterogenous interventions [23]. Importantly, most prior reviews did not specifically evaluate potential effects of school food environment interventions on dietary intakes, and have grouped together highly varied programs potentially leading to biased inferences [26–32]. Our findings extend these results by specifically evaluating school food environment policies and quantifying their effects on dietary intakes, as well as separately evaluating direct provision, competitive food/beverage standards, and school meal standards with careful consideration of potential heterogeneity. We also looked for sustainability: while few studies were identified, the results suggested that dietary improvements are difficult to sustain if school food environment policies are cancelled.

Our evaluation has several strengths. Evidence was based on interventions, most of which were randomized, increasing reliance in validity of results. We evaluated changes in diet, adiposity, and metabolic risk factors, providing a more coherent and comprehensive picture of the evidence. We focused on habitual (within and outside school) dietary intakes, rather than in-school intake alone. A systematic search of multiple databases made it less likely that major relevant reports were missed. Standardized methods and analytic techniques and duplicate full text reviews and data extractions reduced errors and bias. Standardization of interventions and outcomes facilitated quantitative pooling. We explored multiple factors for potential modifying effects.

Potential limitations should be considered. Educational systems and schools vary within and across nations, which could contribute to unmeasured heterogeneity. Intensity or success of policy implementation could modify results, but these are difficult to quantify; e.g., due to varying professional education or technical assistance for food service directors; differences in how schools prepare, offer, sell, serve, or purchase food; and policy nutritional guidelines. Most studies did not report details by socioeconomic indicators, which could modify efficacy of some programs. Costs and cost-effectiveness were generally not reported. Several studies included other intervention components that might contribute to impact. Some studies were judged to have lower quality scores, that could weaken or bias results. Evaluation of heterogeneity and publication bias is dependent on total numbers of studies, limiting statistical power for some endpoints. Most studies were from high-income Western countries, highlighting the need for research in lower-income nations.

In conclusion, this systematic review and meta-analysis demonstrates that specific school food environment policy interventions can improve targeted dietary behaviors. These findings inform ongoing policy discussions and debates on best practices to improve childhood dietary habits and health.

## Supporting information

S1 File Supplementary material. **Appendix A.** PRISMA Checklist. **Appendix B.** Study protocol. **Appendix C.** Search query for PubMed/ Medline. **Appendix D.** Statistical Analysis. **Table A.** Quality Assessment Criteria. **Table B.** Meta-analyses of randomized and quasi-experimental interventions evaluating school food environment policies and dietary habits or adiposity in children. **Table C.** Prespecified sources of heterogeneity explored among interventions evaluating the effect of competitive food and beverage standards in schools on dietary intakes or adiposity in children. **Table D.** Prespecified sources of heterogeneity explored among interventions evaluating the effect of school meal standards on dietary intakes or meal contents in children. **Figure A.** Effect of direct provision of fruits and vegetables in schools on fruit intake in children by prespecified sources of heterogeneity. **Figure B.** Effect of direct provision of fruits and vegetables in schools on vegetable intake in children by prespecified sources of heterogeneity. **Figure C.** Effect of direct provision of fruits and vegetables in schools on fruit and vegetable intake in children by prespecified sources of heterogeneity. **Figure D.** Effect of competitive food and beverage standards in schools on sugar-sweetened beverages and unhealthy snack intake in children by prespecified sources of heterogeneity. **Figure E.** Effect of school meal standards in schools on total fat intake in children by prespecified sources of heterogeneity. **Figure F.** Effect of school meal standards in schools on saturated fat intake in children by prespecified sources of heterogeneity. **Figure G.** Effect of competitive food and beverage standards in schools on overweight and obesity prevalence in children. **Figure H.** Effect of competitive food and beverage standards in schools on odds of overweight and obesity in children. **Figure I.** Effect of competitive food and beverage standards in schools on BMI in children. **Figure J.** Effect of competitive food and beverage standards in schools on BMI z-score in children. **Figure K.** Effect of school meal standards on total fat intake or meal content in children. **Figure L.** Effect of school meal standards on saturated fat intake or meal content in children. **Figure M.** Effect of school meal standards on total caloric intake or meal content in children. **Figure N.** Effect of school meal standards on sodium intake or meal content in children. **Figure O.** Begg’s funnel plots for graphical evaluation of potential publication bias for the effect of direct provision of fruits and vegetables in schools on fruit, vegetable and caloric intake in children. **Figure P.** Begg’s funnel plots for graphical evaluation of potential publication bias for the effect of competitive food and beverage standards in schools on dietary intakes or adiposity in children. **Figure Q.** Begg’s funnel plots for graphical evaluation of potential publication bias for the effect of school meal standards on dietary intakes or meal contents in children.
(DOCX)
