# Supplementary material for: Effectiveness of school food environment policies on children’s dietary behaviors: A systematic review and meta-analysis
Source: PLoS One. 2018 Mar 29;13(3):e0194555. doi: 10.1371/journal.pone.0194555 (PMC5875768; doi:10.1371/journal.pone.0194555)
Supplement: S1 File — Appendix A. PRISMA Checklist. Appendix B. Study protocol. Appendix C. Search query for PubMed/ Medline. Appendix D. Statistical Analysis. Table A. Quality Assessment Criteria. Table B. Meta-analyses of randomized and quasi-experimental interventions evaluating school food environment policies and dietary habits or adiposity in children. Table C. Prespecified sources of heterogeneity explored among interventions evaluating the effect of competitive food and beverage standards in schools on dietary intakes or adiposity in children. Table D. Prespecified sources of heterogeneity explored among interventions evaluating the effect of school meal standards on dietary intakes or meal contents in children. Figure A. Effect of direct provision of fruits and vegetables in schools on fruit intake in children by prespecified sources of heterogeneity. Figure B. Effect of direct provision of fruits and vegetables in schools on vegetable intake in children by prespecified sources of heterogeneity. Figure C. Effect of direct provision of fruits and vegetables in schools on fruit and vegetable intake in children by prespecified sources of heterogeneity. Figure D. Effect of competitive food and beverage standards in schools on sugar-sweetened beverages and unhealthy snack intake in children by prespecified sources of heterogeneity. Figure E. Effect of school meal standards in schools on total fat intake in children by prespecified sources of heterogeneity. Figure F. Effect of school meal standards in schools on saturated fat intake in children by prespecified sources of heterogeneity. Figure G. Effect of competitive food and beverage standards in schools on overweight and obesity prevalence in children. Figure H. Effect of competitive food and beverage standards in schools on odds of overweight and obesity in children. Figure I. Effect of competitive food and beverage standards in schools on BMI in children. Figure J. Effect of competitive food and beverage standards in schools on BM [file pone.0194555.s001.docx]

**SUPPLEMENTARY MATERIAL**

**Effectiveness of School Food Environment Policies on Children’s Dietary Behaviors: A Systematic Review and Meta-Analysis**

Micha R, Karageorgou D, Bakogianni I, Trichia E, Whitsel LP, Story M, Peñalvo JL, Mozaffarian D

[**Appendix A.** PRISMA Checklist. 3](#_Toc509418888)

[**Appendix B.** Study protocol. 5](#_Toc509418889)

[**Appendix C.** Search query for PubMed/ Medline. 8](#_Toc509418890)

[**Appendix D.** Statistical Analysis. 10](#_Toc509418891)

[**Table A.** Quality Assessment Criteria. 11](#_Toc509418892)

[**Table B.** Meta-analyses of randomized and quasi-experimental interventions evaluating school food environment policies and dietary habits or adiposity in children. 12](#_Toc509418893)

[**Table C.** Prespecified sources of heterogeneity explored among interventions evaluating the effect of competitive food and beverage standards in schools on dietary intakes or adiposity in children. 14](#_Toc509418894)

[**Table D.** Prespecified sources of heterogeneity explored among interventions evaluating the effect of school meal standards on dietary intakes or meal contents in children. 15](#_Toc509418895)

[**Figure A.** Effect of direct provision of fruits and vegetables in schools on fruit intake in children by prespecified sources of heterogeneity. 16](#_Toc509418896)

[**Figure B.** Effect of direct provision of fruits and vegetables in schools on vegetable intake in children by prespecified sources of heterogeneity. 17](#_Toc509418897)

[**Figure C.** Effect of direct provision of fruits and vegetables in schools on fruit and vegetable intake in children by prespecified sources of heterogeneity. 18](#_Toc509418898)

[**Figure D.** Effect of competitive food and beverage standards in schools on sugar-sweetened beverages and unhealthy snack intake in children by prespecified sources of heterogeneity. 19](#_Toc509418899)

[**Figure E.** Effect of school meal standards in schools on total fat intake in children by prespecified sources of heterogeneity. 20](#_Toc509418900)

[**Figure F.** Effect of school meal standards in schools on saturated fat intake in children by prespecified sources of heterogeneity. 21](#_Toc509418901)

[**Figure G.** Effect of competitive food and beverage standards in schools on overweight and obesity prevalence in children. 22](#_Toc509418902)

[**Figure H.** Effect of competitive food and beverage standards in schools on odds of overweight and obesity in children. 23](#_Toc509418903)

[**Figure I.** Effect of competitive food and beverage standards in schools on BMI in children. 24](#_Toc509418904)

[**Figure J.** Effect of competitive food and beverage standards in schools on BMI z-score in children. 25](#_Toc509418905)

[**Figure K.** Effect of school meal standards on total fat intake or meal content in children. 26](#_Toc509418906)

[**Figure L.** Effect of school meal standards on saturated fat intake or meal content in children. 27](#_Toc509418907)

[**Figure M.** Effect of school meal standards on total caloric intake or meal content in children. 28](#_Toc509418908)

[**Figure N.** Effect of school meal standards on sodium intake or meal content in children. 29](#_Toc509418909)

[**Figure O.** Begg’s funnel plots for graphical evaluation of potential publication bias for the effect of direct provision of fruits and vegetables in schools on fruit, vegetable and caloric intake in children. 30](#_Toc509418910)

[**Figure P.** Begg’s funnel plots for graphical evaluation of potential publication bias for the effect of competitive food and beverage standards in schools on dietary intakes or adiposity in children. 31](#_Toc509418911)

[**Figure Q.** Begg’s funnel plots for graphical evaluation of potential publication bias for the effect of school meal standards on dietary intakes or meal contents in children. 32](#_Toc509418912)

[**References** 33](#_Toc509418913)

# Appendix A. PRISMA Checklist.

| **Section/topic** | **#** | **Checklist item** | **Reported on** |
| --- | --- | --- | --- |
| **TITLE** | | |  |
| Title | 1 | Identify the report as a systematic review, meta-analysis, or both. | Title page |
| **ABSTRACT** | | |  |
| Structured summary | 2 | Provide a structured summary including, as applicable: background; objectives; data sources; study eligibility criteria, participants, and interventions; study appraisal and synthesis methods; results; limitations; conclusions and implications of key findings; systematic review registration number. | Abstract |
| **INTRODUCTION** | | |  |
| Rationale | 3 | Describe the rationale for the review in the context of what is already known. | Introduction |
| Objectives | 4 | Provide an explicit statement of questions being addressed with reference to participants, interventions, comparisons, outcomes, and study design (PICOS). | Introduction |
| **METHODS** | | |  |
| Protocol and registration | 5 | Indicate if a review protocol exists, if and where it can be accessed (e.g., Web address), and, if available, provide registration information including registration number. | Appendix B in S1 File – Study protocol |
| Eligibility criteria | 6 | Specify study characteristics (e.g., PICOS, length of follow-up) and report characteristics (e.g., years considered, language, publication status) used as criteria for eligibility, giving rationale. | Primary exposures and outcomes; study selection; study protocol |
| Information sources | 7 | Describe all information sources (e.g., databases with dates of coverage, contact with study authors to identify additional studies) in the search and date last searched. | Search strategy; study protocol |
| Search | 8 | Present full electronic search strategy for at least one database, including any limits used, such that it could be repeated. | Search query for PubMed/ Medline |
| Study selection | 9 | State the process for selecting studies (i.e., screening, eligibility, included in systematic review, and, if applicable, included in the meta-analysis). | Search strategy; study protocol |
| Data collection process | 10 | Describe method of data extraction from reports (e.g., piloted forms, independently, in duplicate) and any processes for obtaining and confirming data from investigators. | Data extraction; study protocol |
| Data items | 11 | List and define all variables for which data were sought (e.g., PICOS, funding sources) and any assumptions and simplifications made. | Data extraction; study protocol |
| Risk of bias in individual studies | 12 | Describe methods used for assessing risk of bias of individual studies (including specification of whether this was done at the study or outcome level), and how this information is to be used in any data synthesis. | Data extraction; Table A in, S1 File; statistical analysis |
| Summary measures | 13 | State the principal summary measures (e.g., risk ratio, difference in means). | Statistical analysis |
| Synthesis of results | 14 | Describe the methods of handling data and combining results of studies, if done, including measures of consistency (e.g., I^2^) for each meta-analysis. | Statistical analysis |
| Risk of bias across studies | 15 | Specify any assessment of risk of bias that may affect the cumulative evidence (e.g., publication bias, selective reporting within studies). | Statistical analysis |
| Additional analyses | 16 | Describe methods of additional analyses (e.g., sensitivity or subgroup analyses, meta-regression), if done, indicating which were pre-specified. | Statistical analysis |
| **RESULTS** | | |  |
| Study selection | 17 | Give numbers of studies screened, assessed for eligibility, and included in the review, with reasons for exclusions at each stage, ideally with a flow diagram. | Figure 1 |
| Study characteristics | 18 | For each study, present characteristics for which data were extracted (e.g., study size, PICOS, follow-up period) and provide the citations. | Table 1 |
| Risk of bias within studies | 19 | Present data on risk of bias of each study and, if available, any outcome level assessment (see item 12). | Table 1 |
| Results of individual studies | 20 | For all outcomes considered (benefits or harms), present, for each study: (a) simple summary data for each intervention group (b) effect estimates and confidence intervals, ideally with a forest plot. | Figures 2-4; Figures G-N in S1 File |
| Synthesis of results | 21 | Present the main results of the review. If meta-analyses are done, include for each, confidence intervals and measures of consistency. | Figures 2-4; Figures G-N in S1 File; Table B in S1 File |
| Risk of bias across studies | 22 | Present results of any assessment of risk of bias across studies (see Item 15). | Figures O-Q in S1 File |
| Additional analysis | 23 | Give results of additional analyses, if done (e.g., sensitivity or subgroup analyses, meta-regression [see Item 16]). | Table 2; Tables C and D in S1 File; Figures A-F in S1 File |
| **DISCUSSION** | | |  |
| Summary of evidence | 24 | Summarize the main findings including the strength of evidence for each main outcome; consider their relevance to key groups (e.g., healthcare providers, users, and policy makers). | Discussion, 1^st^ paragraph |
| Limitations | 25 | Discuss limitations at study and outcome level (e.g., risk of bias), and at review-level (e.g., incomplete retrieval of identified research, reporting bias). | Discussion, 8^th^ paragraph |
| Conclusions | 26 | Provide a general interpretation of the results in the context of other evidence, and implications for future research. | Discussion, conclusions |
| **FUNDING** | | |  |
| Funding | 27 | Describe sources of funding for the systematic review and other support (e.g., supply of data); role of funders for the systematic review. | Funding; role of funding source |

*From:*  Moher D, Liberati A, Tetzlaff J, Altman DG, The PRISMA Group (2009). Preferred Reporting Items for Systematic Reviews and Meta-Analyses: The PRISMA Statement. PLoS Med 6(7): e1000097. doi:10.1371/journal.pmed1000097

For more information, visit: **www.prisma-statement.org**.

# Appendix B. Study protocol.

**Title**

Effectiveness of School Food Environment Policies on Children’s Dietary Behaviors: A Systematic Review and Meta-Analysis

**Objective**

To systematically review and quantify the impact of school food environment policy interventions on dietary habits, adiposity, and metabolic risk in children.

**Methods**

The recommendations of Preferred Reporting Items for Systematic reviews and Meta-Analyses (PRISMA) guidelines were followed for clinical trials (randomized and quasi-experimental), during all stages of the design, implementation, and reporting of this meta-analysis.

**Definition of Exposure/ Intervention**

- Implementation of school food environment policies (including procurement policies) on types of food/ beverage or food/ beverage content targeted to improve general health, at school and of at least 4 weeks duration.
- If multi-component intervention, we included only if food/beverage environmental policy is a major component of the intervention (qualitatively, <30%, as judged by two independent reviewers).
- Not included/ unrelated interventions:
  - Policies targeted at health and safety issues (e.g., epidemic restrictions like salmonella, encephalopathy, water sanitation, additives, coloring, etc).
  - Food fortification targeted at micronutrient, mineral, and vitamin deficiencies (e.g., salt iodination).
  - Food/ menu labeling policies.
  - Economic incentives – taxation, subsidies (e.g., vouchers).
  - Restrictions on advertisements for less healthful foods/ beverages or increased advertisements for healthful foods/ beverages.
  - Education regulations/ laws/ campaigns (e.g., teaching nutrition in schools, school gardens).
  - Legislation on food/ crop production (e.g., milk, fish, meat, sugar, poultry) or agricultural policies.
  - Body mass index (BMI) regulation (e.g., BMI report cards).
  - Any alcohol regulation.

**Definition of Outcome(s)**

- Primary Outcome
  - Change (continuous) in targeted dietary intake: including foods, beverages, and nutrients corresponding to overall intake or proxy of overall intake (e.g., fruits, vegetables, milk, sugar-sweetened beverages, meats, dietary fiber, salt/ sodium, total fat, saturated fat).
  - Change (continuous) in sales/purchases data (use as a proxy for difference in dietary consumption).
- Secondary Outcomes
  - Change (continuous) in in-school meal nutrient content.
  - Change (continuous) in-school meal intake.
  - Change (continuous)in total caloric intake.
  - Change (continuous) in adiposity measures (e.g., body mass index, overweight/obesity).
  - Change (continuous) in metabolic measures (e.g., blood lipids, blood glucose, blood pressure).

**Inclusion Criteria**

1. **Study Design:** All interventions (randomized clinical trials or quasi-experimental) that assessed the impact of school food environment policies on dietary habits, adiposity or metabolic risk factors among generally healthy children.
2. **Follow-up Duration:** ≥4 weeks. For outcomes evaluated at multiple time points, we extracted data on the latest follow-up measure at end-intervention.
3. **Sustainability Duration:** ≥4 weeks. For outcomes evaluated additionally after the end of the intervention, we extracted data on the latest follow-up measure after the end of the intervention.
4. **Population:** Children.
5. **Setting:** Preschool, primary, or secondary schools.
6. **Exposure:** Implementation of school food environment policies (provision of healthful foods/beverages, competitive food/beverage standards, school meal standards) on types of food/ beverage or food/ beverage content targeted to improve general health, including procurement policies. Multi-component interventions were included if the school food environmental policy is a major component of the intervention (at least 30% of the intervention, as judged by two independent reviewers).
7. **Outcome:** Intake of foods/ beverages, sales/ purchases of foods/ beverages, caloric intake, adiposity, metabolic risk factors, meal content.
8. **Effect Estimate:** Studies providing a quantitative change in the outcome.
9. **Language:** No restrictions.
10. **Publication Type:** Full-text, published, peer reviewed.
11. **Year:** Earliest available through Dec 14, 2017.

**Exclusion Criteria**

1. **Study Design:** Observational studies (single period cross-sectional studies, retrospective studies, case-control studies), modeling studies, laboratory experiments, reviews, commentaries, methodology papers, books, and studies for which full-text articles could not be retrieved from online searches, journals, or author contact.
2. **Follow-up Duration:** <4 weeks.
3. **Sustainability Duration:** <4 weeks.
4. **Population:** Older than 18 years old, pregnant women, and specified diseased or special (e.g., vegetarians vs non-vegetarians) populations.
5. **Setting:** Any other than school (e.g., workplace, hospital).
6. **Exposure:** Unrelated interventions as specified above.
7. **Outcome:** Non-targeted outcomes, with the exception of energy intake and adiposity measures that were extracted whenever available. Knowledge or attitudes.
8. **Duplicate Publications:** When duplicate publications from the same study were identified, the report on the longest follow-up duration was included.

**Data Extraction**

For each study, data were extracted independently and in duplicate using a standardized electronic template (Microsoft Access, Office 2010). Extracted information included first author, publication year, study location, design, population (age, sex, race, sample size), intervention characteristics (components, targets, duration), outcome data including habitual (within and outside school) and in-school (e.g., lunch, breakfast, total in-school) intakes (definition, ascertainment methods, effect size, precision estimate), covariates, and for multi-component interventions, the relative contribution of the food environment policy component to the overall intervention (low: 30-59%, medium: 60-89%, high: ≥90%; qualitatively assessed). Missing data or definitions were resolved by direct contact with authors, where possible.

# Appendix C. Search query for PubMed/ Medline.

**(1) Intervention Query**

(“Government Regulation” [MeSH] OR “Legislation, Food” [MeSH] OR “legislation” [tiab] OR “legislations” [tiab] OR “law” [tiab] OR “laws” [tiab] OR "ban" [tiab] OR "bans" [tiab] OR "mandate" [tiab] OR "mandates" [tiab] OR "nutrition policy" [tiab] OR "restrict" [tiab] OR "school food policy" [tiab] OR ("availability" [tiab] AND "school-based intervention" [tiab]) OR ("availability" [tiab] AND "cafeteria" [tiab]) OR ("availability" [tiab] AND "work" [tiab]) OR ("promoting" [tiab] AND "school environment" [tiab]) OR "cafeteria-based intervention" [tiab] OR "health promotion" [tiab] OR "school-based program" [tiab] OR "food service intervention" [tiab] OR "foodservice intervention" [tiab] OR "foodservice policy" [tiab] OR "food service policy" [tiab] OR "foodservice program" [tiab] OR "foodservice programs" [tiab] OR procurement[tiab] OR procurements[tiab])

**AND**

(("Health Policy" [MeSH] OR "Restaurants/legislation and jurisprudence" [MeSH] OR "Health Behavior" [MeSH] OR "Environmental Policy" [MeSH] OR "Nutrition Policy" [MeSH] OR "Obesity/epidemiology" [MeSH] OR "School Health Services/standards" [MeSH] OR "Health Promotion" [MeSH] OR "Carbonated Beverages/statistics and numerical data" [MeSH] OR "Schools/legislation & jurisprudence" [MeSH] OR "Food Services/standards" [MeSH] OR "Food Industry/legislation & jurisprudence" [MeSH] OR "Food Services/legislation & jurisprudence" [MeSH] OR "Dietary Fats/standards" [MeSH]))

**AND**

**(2) Diet Query**

(“diet” [MeSH] OR “diets” [tiab] OR “food” [tiab] OR “foods” [tiab] OR “nutrition” [tiab] OR "nutrient" [tiab] OR "nutrients" [tiab] OR "beverages” [MeSH] OR “beverage” [tiab] OR “beverages” [tiab] OR “soda” [tiab] OR “fruit” [tiab] OR “fruits”[tiab] OR “vegetable”[tiab] OR “vegetables”[tiab] OR “meat” [tiab] OR "meat products" [tiab] OR “dairy” [tiab] OR “candy” [tiab] OR “sweet”[tiab] OR “sweets”[tiab] OR “Fatty Acids” [MeSH] OR "fat" [tiab] OR "fats" [tiab] OR "fatty acids" [tiab] OR "oil" [tiab] OR "oils" [tiab] OR "unsaturated" [tiab] OR "saturated" [tiab] OR "trans fatty" [tiab] OR "trans unsaturated" [tiab] OR "trans fat" [tiab] OR "trans fats" [tiab] OR "sodium, dietary" [MeSH] OR "sodium" [tiab] OR “salt” [tiab] OR "lunch" [tiab] OR "lunches" [tiab] OR breakfast [tiab] OR "meal" [tiab] OR "meals" [tiab] OR "water" [tiab] OR “sugar” [tiab])

**AND**

**(3) Outcome Query**

(“eating” [tiab] OR “intake” [tiab] OR “consumption” [tiab] OR "intakes" [tiab] OR "availability" [tiab] OR "offering" [tiab] OR "offerings" [tiab] OR “coronary heart disease” [tiab] OR “Cardiovascular Diseases” [MeSH] OR “Cardiovascular Disease” [tiab] OR “Cardiovascular Diseases” [tiab] OR “Hypertension” [tiab] OR “Diabetes” [tiab] OR “Obesity” [MeSH] OR “Obesity” [tiab] OR “Overweight” [MeSH] OR “Overweight” [tiab] OR "Body Mass Index" [MeSH] OR “Cholesterol” [tiab] OR “food formulation” [tiab] OR “product formulation” [tiab] OR "sales" [tiab] OR "revenue" [tiab] OR "revenues" [tiab] OR "percentage of calories" [tiab] OR "percentages of calories" [tiab] OR "purchase" [tiab] OR "purchases" [tiab] or "glucose" [tiab] OR "BMI" [tiab] OR "Body Mass Index" [tiab] OR "blood pressure" [tiab] OR "blood pressure" [MeSH])

**AND**

**(4) Location Query**

("country" [tiab] OR "countries" [tiab] OR "nation" [tiab] OR "National" [tiab] OR "nation-wide" [tiab] OR "state" [tiab] OR "school" [tiab] OR "schools" [tiab] OR "district" [tiab] OR "worksite" [tiab] OR "work" [tiab] OR "local" [tiab] OR "region" [tiab] OR "region*" [tiab] OR "city" [tiab] OR "cities" [tiab] OR "province" [tiab] OR "county" [tiab] OR "statewide" [tiab] OR "citywide" [tiab] OR "workplace" [tiab] OR "cafeteria" [tiab] OR "restaurant" [tiab] OR "supermarket" [tiab] OR "store" [tiab] OR "shop" [tiab])

**NOT**

("Soil" [tiab] OR "spectrometry" [tiab] OR "antibiotic" [tiab] OR "antibiotics" [tiab] OR "simulation" [tiab] OR "safety" [tiab] OR "chromatography" [tiab] OR "pollution" [tiab] OR "pathology" [tiab] OR "phosphate" [tiab] OR "gene" [tiab] OR "livestock" [tiab] OR "pig" [tiab] OR "pig*" [tiab])

Abstracts identified: 1502 Hits

Date: 12/14/2017

# Appendix D. Statistical Analysis.

**Effect Size and Standard Error Estimation**

Study-specific effect sizes were pooled using inverse-variance weighted random-effects meta-analysis (metan command in Stata). The primary outcome was the mean continuous change at follow-up adjusted for baseline values and relevant covariates if necessary. For interventions with an external control, the primary effect was the change between groups; for quasi-experimental studies with no control group, the primary effect was the within-group change. The uncertainty (standard error, SE) of the effect size was extracted or calculated based on other statistics (e.g., 95% confidence intervals (CIs), standard deviation (SD), P value). For two studies,^1,2^ the SE was not reported nor could be computed, and was estimated conservatively as the largest observed SE from other studies. If only an upper bound p-value was reported (e.g., p>0.05) we used this upper bound to estimate SE in main analysis and additional ranges in sensitivity analyses. If only a lower bound p-value was reported (e.g., p<0.05), we used the mid-point between zero and that lower bound (e.g., 0.025) to estimate the SE. If sufficient data were not available to calculate the SE by any of these approaches, we replaced it with the highest SE of all studies included in the meta-analysis as a conservative approach. If the estimation included paired observations and the sample covariance was not reported, we used a correlation of 0.5 for main analysis and 0.1 and 0.9 for sensitivity analyses. In addition to continuous effect sizes, we extracted other relevant effect sizes (e.g., percentage meeting a cutpoint, odds ratio, ratio of the means, other relative changes) and their statistical uncertainty. Separate intervention arms or outcomes from the same study were included as separate estimates in the meta-analyses; subgroup findings from the same intervention arm or outcome (e.g., by sex, age) were first combined using study-specific meta-analysis.

We separately pooled findings for direct provision of healthful foods/ beverages, competitive food/beverage standards, and school meal standards. Effect sizes were standardized to consistent units: e.g., 80 g serving/d for F&V, 12-oz serving/d for SSBs, kcal/d for calories, % energy (E)/d, g/d or mg/d for nutrients, and kg/m^2^ or z-score for BMI. Endpoints that could not be standardized (e.g., consumption expressed as a score, proportion of children consuming a given level) or separately meta-analyzed were included in qualitative assessment of the evidence. When multiple overlapping outcomes were reported (e.g., fruit with vs. without 100% juice), we extracted the outcome mostly closely aligned to a standardized definition, e.g. total fruits (fresh, raw, canned, or dried), excluding fruit juice; total vegetables, excluding white potatoes; and total SSBs (soda, energy drinks, sweetened teas, etc.). For studies reporting subcomponents of these definitions (e.g., separate subtypes of vegetables, of sweet snacks, F&V separately), we first summed these subtypes.

# Table A. Quality Assessment Criteria.

| **Criterion** | **Range** | **Description** | |
| --- | --- | --- | --- |
| Design | 0-1 | 1 | if randomized trial |
|  |  | 0 | if quasi-experimental design of any kind |
| Assessment of  intervention/exposure | 0-1 | 1 | if the intervention/exposure has been clearly defined and measured |
|  |  | 0 | if the intervention/exposure has not been clearly defined and measured |
| Assessment of outcome | 0-1 | 1 | if the outcome has been clearly defined and measured |
|  |  | 0 | if the outcome definition and measurement has not been clearly described |
| Control for confounding | 0-1 | 1 | if RCT or sufficient/ appropriate control for major confounders |
|  |  | 0 | if insufficient control for major confounders |
| Evidence of selection bias | 0-1 | 1 | if absence of evidence for selection bias |
|  |  | 0 | if substantial presence of evidence for selection bias |

Each criterion received a score of 1 or 0 (1 being better), and an overall quality score was calculated as the sum of individual scores; with 0-3 considered lower quality and 4-5, higher quality.

# Table B. Meta-analyses of randomized and quasi-experimental interventions evaluating school food environment policies and dietary habits or adiposity in children.

| **Strategy/ Dietary Target** | **Intake/ Content** | **Studies in Meta-analysis^a^** | **Unit of Change** | **Mean Change  (95% CI) ^b^** |
| --- | --- | --- | --- | --- |
| **Direct Provision of Healthful Foods and Beverages** | | | | |
| Fruits | Habitual intake ^c, d^ | 15 (15) | servings (80 g)/d | 0.27 (0.17, 0.36) |
|  | Habitual intake - Sustainability | 3 (3) | servings (80 g)/d | -0.18 (-0.51, 0.15) |
|  | In-school total intake ^c^ | 5 (5) | servings (80 g)/d | 0.27 (0.13, 0.42) |
| Vegetables | Habitual intake ^c, d^ | 11 (11) | servings (80 g)/d | 0.04 (0.01, 0.08) |
|  | In-school total intake ^c^ | 3 (3) | servings (80 g)/d | 0.03 (-0.06, 0.11) |
| Fruits and vegetables | Habitual intake ^c, d^ | 16 (16) | servings (80 g)/d | 0.28 (0.17, 0.40) |
|  | In-school total intake ^c, d^ | 6 (6) | servings (80 g)/d | 0.38 (0.23, 0.53) |
| Total calories | Habitual intake ^d^ | 6 (6) | kcal/d | -56 (-174, 62) |
| Water | Habitual intake | 3 (3) | glasses (8-oz)/d | 0.33 (-0.27, 0.93) |
| Obesity |  | 2 (2) | odds ratio | 1.25 (1.07, 1.46) |
| Overweight/ Obesity |  | 2 (2) | odds ratio | 1.04 (0.91, 1.19) |
| BMI |  | 3 (3) | kg/m^2^ | 0.19 (-0.12, 0.50) |
|  |  | 2 (2) | z-score | 0.01 (-0.04, 0.05) |
| **Competitive Food and Beverage Standards** | | |  |  |
| Sugar-sweetened beverages | Habitual intake ^c^ | 3 (3) | servings (12-oz)/d | -0.18 (-0.31, -0.05) |
|  | In-school total intake ^c, d^ | 4 (5) | servings (12-oz)/d | -0.02 (-0.04, 0.01) |
| Unhealthy snacks | Habitual intake ^c^ | 2 (3) | servings/d | -0.17 (-0.22, -0.13) |
|  | In-school total intake ^c, d^ | 4 (9) | servings/d | -0.05 (-0.08, -0.02) |
| Total calories | Habitual intake ^d^ | 5 (5) | kcal/d | -79 (-179, 21) |
|  | In-school lunch intake | 3 (3) | kcal/d | -49 (-196, 99) |
| Total fat | Habitual intake | 3 (3) | %E/d | -1.94 (-2.27, -1.61) |
|  | In-school lunch intake | 2 (2) | %E/d | -8.32 (-13.13, -3.51) |
|  | Habitual intake | 3 (3) | g/d | -8.18 (-15.54, -0.82) |
|  | In-school lunch intake | 3 (3) | g/d | -0.55 (-3.31, 2.22) |
| Saturated fat | In-school lunch intake | 2 (2) | g/d | -0.20 (-2.10, 1.70) |
| Overweight |  | 5 (6) | prevalence % ^c, d^ | -0.95 (-2.23, 0.33) |
|  |  | 4 (6) | odds ratio ^c^ | 0.93 (0.86, 1.02) |
| Obesity |  | 9 (10) | prevalence % ^c, d^ | -1.02 (-2.56, 0.52) |
|  |  | 6 (8) | odds ratio ^c, d^ | 0.99 (0.93, 1.06) |
| Overweight/ Obesity |  | 5 (5) | prevalence % ^c, d^ | 0.24 (-0.54, 1.02) |
|  |  | 2 (2) | odds ratio ^c^ | 1.01 (0.84, 1.21) |
| BMI |  | 5 (6) | kg/m^2 c, d^ | -0.06 (-0.41, 0.28) |
|  |  | 5 (5) | z-score ^c, d^ | -0.01 (-0.03, 0.02) |
| **School Meal Standards** |  |  |  |  |
| Fruits | Habitual intake | 2 (2) | servings (80 g)/d | 0.76 (0.37, 1.16) |
|  | In-school lunch intake | 4 (4) | servings (80 g)/d | 0.01 (-0.06, 0.07) |
| Vegetables | Habitual intake | 2 (2) | servings (80 g)/d | 0.30 (-0.001, 0.59) |
|  | In-school lunch intake | 4 (4) | servings (80 g)/d | 0.003 (-0.11, 0.12) |
| Fruits and vegetables | Habitual intake ^d^ | 5 (5) | servings (80 g)/d | 0.12 (-0.08, 0.31) |
|  | In-school lunch intake ^d^ | 5 (5) | servings (80 g)/d | 0.00 (-0.12, 0.12) |
| Total fat | Habitual intake ^c, d^ | 6 (6) | %E/d | -1.49 (-2.42, -0.57) |
|  | In-school lunch intake ^c, d^ | 9 (9) | %E/d | -8.27 (-10.03, -6.52) |
|  | In-school meal intake ^e^ | 9 (10) | %E/d | -7.12 (-9.48, -4.75) |
|  | Habitual intake ^c, d^ | 6 (6) | g/d | -5.72 (-10.89, -0.54) |
|  | In-school lunch intake ^c, d^ | 9 (9) | g/d | -3.80 (-5.34, -2.26) |
|  | In-school meal intake ^e^ | 9 (10) | g/d | -3.71 (-5.20, -2.22) |
|  | In-school lunch content ^c, d^ | 10 (11) | g/lunch | -3.35 (-5.20, -1.50) |
|  | In-school breakfast content | 3 (4) | g/breakfast | -1.93 (-6.72, 2.87) |
|  | In-school meal content ^e^ | 11 (15) | g/meal | -2.97 (-4.78, -1.16) |
| Saturated fat | Habitual intake ^c^ | 4 (4) | %E/d | -0.93 (-1.15, -0.70) |
|  | In-school lunch intake ^c, d^ | 9 (9) | %E/d | -2.75 (-4.39, -1.11) |
|  | In-school meal intake ^e^ | 9 (10) | %E/d | -2.46 (-4.04, -0.89) |
|  | Habitual intake ^c^ | 4 (4) | g/d | -2.78 (-5.55, -0.004) |
|  | In-school lunch intake ^c, d^ | 9 (9) | g/d | -2.09 (-2.52, -1.66) |
|  | In-school meal intake ^e^ | 9 (10) | g/d | -1.82 (-2.53, -1.11) |
|  | In-school lunch content ^c, d^ | 7 (8) | g/lunch | -1.27 (-1.85, -0.68) |
|  | In-school meal content ^e^ | 8 (11) | g/meal | -1.27 (-1.94, -0.59) |
| Total calories | Habitual intake ^c, d^ | 8 (8) | kcal/d | -38 (-137, 62) |
|  | In-school lunch intake ^c, d^ | 11 (11) | kcal/d | -28 (-76, 20) |
|  | In-school meal intake ^e^ | 11 (12) | kcal/d | -29 (-76, 18) |
|  | In-school lunch content ^c, d^ | 9 (10) | kcal/lunch | -56 (-76, -36) |
|  | In-school breakfast content | 3 (4) | kcal/breakfast | -73 (-224, 78) |
|  | In-school meal content ^e^ | 10 (14) | kcal/meal | -57 (-98, -16) |
| Sodium | Habitual intake ^c^ | 4 (4) | mg/d | -170 (-242, -98) |
|  | In-school lunch intake ^c, d^ | 6 (6) | mg/d | -227 (-384, -69) |
|  | In-school meal intake ^e^ | 6 (7) | mg/d | -221 (-371, -71) |
|  | In-school lunch content ^c, d^ | 6 (7) | mg/lunch | -182 (-283, -81) |
|  | In-school meal content ^e^ | 7 (10) | mg/meal | -172 (-287, -57) |
| Milk/ Milk products | Habitual intake | 2 (2) | cups/d | 0.22 (0.17, 0.28) |
| Fiber | Habitual intake | 3 (3) | g/d | 0.08 (-0.84, 1.00) |
|  | In-school lunch intake | 2 (2) | g/d | 0.55 (-1.90, 3.00) |
|  | In-school lunch content | 2 (2) | g/lunch | 2.07 (-0.66, 4.8) |
|  | In-school meal content ^e^ | 2 (3) | g/meal | 1.09 (0.27, 1.92) |
| Whole grains | Habitual intake | 2 (2) | servings/d | 0.14 (-0.11, 0.39) |
|  | In-school lunch intake | 2 (2) |  | 0.49 (-0.37, 1.35) |
| Total carbohydrates | In-school lunch intake | 2 (3) | %E/d | 8.17 (0.70, 15.65) |
|  | In-school lunch intake | 2 (3) | g/d | 7.42 (-6.01, 20.84) |
| Less “healthy” selections ^g^ | In-school lunch selection ^f^ | 3 (5) | % | -12.10 (-35.14, 10.95) |
| Overweight |  | 2 (2) | prevalence % | -0.30 (-1.79, 1.88) |
| Obesity |  | 3 (3) | prevalence % | -1.06 (-3.80, 1.68) |
|  |  | 2 (2) | odds ratio | 1.02 (0.66, 1.57) |
| Overweight/Obesity |  | 2 (2) | prevalence % | -0.04 (-2.50, 2.43) |
| ΒΜΙ |  | 2 (2) | percentile | -1.01 (-1.62, -0.39) |

^a^ Number of estimates (values in parentheses) can be higher than number of studies if multiple intervention groups or multiple comparisons were available from the same study.

^b^ Study-specific effect sizes were pooled using inverse-variance weighted random-effect models (metan command in STATA). Effect sizes correspond to mean changes standardized across studies to consistent units; and precision estimates to 95% confidence intervals (CIs).

^c^ Meta-analysis plots presented.

^d^ A minimum number of estimates (at least 5) was required to assess heterogeneity.

^e^ School meal refers to lunch and breakfast. Secondary analysis – no heterogeneity sources were assessed.

^f^ Prevalence of students selecting unhealthy snacks at lunch.

^g^ Including desserts, high-fat entrees, starchy foods in oil.

%E=Percentage of energy; BMI=Body Mass Index

# Table C. Prespecified sources of heterogeneity explored among interventions evaluating the effect of competitive food and beverage standards in schools on dietary intakes or adiposity in children.

| **Heterogeneity sources ^a^** | **SSB in-school total intake**  **servings (12-oz)/d** | | **Unhealthy snack in-school total intake servings/d** | | **Obesity prevalence**  **%** | | **BMI  z-score** | |
| --- | --- | --- | --- | --- | --- | --- | --- | --- |
|  | N (n) ^b^ | Mean (95% CI) ^c^ | N (n) ^b^ | Mean (95% CI) ^c^ | N (n) ^b^ | Mean (95% CI) ^c^ | N (n) ^b^ | Mean (95% CI) ^c^ |
| **Overall** | 4 (5) | -0.02 (-0.04, 0.01) | 4 (9) | -0.05 (-0.08, -0.02) | 9 (10) | -1.02, (-2.56, 0.52) | 5 (5) | -0.01 (-0.03, 0.02) |
| **Study design** |  |  |  |  |  |  |  |  |
| RCT | 1 (1) | n/a | 2 (3) | -0.06 (-0.09, -0.02) | 4 (4) | -0.96 (-3.76, 1.83) | 3 (3) | -0.03 (-0.07, 0.00) |
| QED | 3 (4) | -0.02 (-0.7, 0.03) | 2 (6) | -0.05 (-0.10, -0.01) | 5 (6) | -0.97 (-2.86, 0.91) | 2 (2) | 0.01 (0.01, 0.02) |
| **Region** |  |  |  |  |  |  |  |  |
| US/Canada | 4 (5) | Overall ^i^ | 3 (7) | -0.05 (-0.09, -0.01) | 8 (9) | -0.72 (-2.32, 0.89) | 5 (5) | Overall ^i^ |
| Europe/New Zealand | 0 (0) | n/a | 1 (2) | n/a | 1 (1) | n/a | 0 (0) | n/a |
| **Type of intervention ^d^** |  |  |  |  |  |  |  |  |
| Food policy only | 3 (4) | -0.02 (-0.07, 0.03) | 3 (8) | -0.05 (-0.09, -0.01) | 4 (5) | -1.62 (-3.04, -0.20) | 2 (2) | 0.01 (0.01, 0.02) |
| Multi-component | 1 (1) | n/a | 1 (1) | n/a | 5 (5) | -0.21 (-2.64, 2.22) | 3 (3) | -0.03 (-0.07, 0.00) |
| **Non-dietary targets ^e^** |  |  |  |  |  |  |  |  |
| No | 4 (5) | Overall ^j^ | 4 (9) | Overall ^i^ | 6 (7) | -0.75 (-2.63, 1.13) | 3 (3) | 0.01 (0.01, 0.02) |
| Yes |  |  |  |  | 3 (3) | -1.71 (-4.07, 0.66) | 2 (2) | -0.03 (-0.07, 0.00) |
| **Νο of environmental strategies ^f^** | |  |  |  |  |  |  |  |
| 1 | 2 (3) | -0.01 (-0.04, 0.02) | 1 (4) | -0.02 (-0.04, 0.003) | 5 (6) | -1.38 (-3.08, 0.32) | 3 (3) | 0.01 (0.01, 0.02) |
| >1 | 2 (2) | -0.15 (-0.45, 0.15) | 3 (5) | -0.07 (-0.10, -0.03) | 4 (4) | -0.61 (-3.29, 2.07) | 2 (2) | -0.04 (-0.08, -0.003) |
| **School level ^g^** |  |  |  |  |  |  |  |  |
| Primary | 1 (2) | n/a | 2 (6) | -0.03 (-0.07, 0,00) | 5 (6) | -1.11 (-3.53, 1.31) | 1 (1) | n/a |
| Secondary | 2 (2) | -0.14 (-0.46, 0.20) | 1 (2) | n/a | 2 (2) | -1.84 (-4.13, 0.45) | 1 (1) | n/a |
| Primary & secondary | 1 (1) | n/a | 1 (1) | n/a | 2 (2) | 0.80 (-5.85, 7.46) | 3 (3) | 0.01 (0.01, 0.02) |
| **Quality score ^h^** |  |  |  |  |  |  |  |  |
| Low | 3 (4) | -0.02 (-0.07, 0.03) | 2 (6) | -0.05 (-0.10, -0.01) | 5 (6) | -0.97 (-2.86, 0.91) | 2 (2) | 0.01 (0.01, 0.02) |
| High | 1 (1) | n/a | 2 (3) | -0.06 (-0.09, -0.02) | 4 (4) | -0.96 (-3.76, 1.83) | 3 (3) | -0.03 (-0.07, 0.00) |

^a^ Results are presented for selected heterogeneity sources (common across the three strategies of school food environmental policies identified (Table 2, Table D in S1 File) for the outcomes with the largest numbers of estimates. For all other outcomes not presented, no significant heterogeneity sources were identified. None of the identified differences by subgroups were statistically significant by meta-regression (P-heterogeneity>0.05 each.

^b^ Number of estimates (values in parentheses) can be higher than number of studies included in the meta-analyses if multiple intervention groups or multiple comparisons were available from the same study.

^c^ Study-specific effect sizes were pooled using stratified inverse-variance weighted random-effect models (metan command in Stata). Effect sizes correspond to mean changes standardized across studies to consistent units; and precision estimates to 95% confidence intervals (CIs).

^d^ Single-component interventions consisted only of the school food environment policy. Multi-component interventions were included only if the food environment policy was a major component, judged qualitatively to be at least 30% of the overall intervention. Additional potential components included education, food/menu labeling, etc. (see Table 1).

^e^ In addition to the dietary targets, specific interventions also targeted non-dietary targets, such as physical activity and smoking.

^f^ School food environment policy strategies included direct provision of healthful foods, quality standards for competitive foods/ beverages, and quality standards for school meals.

^g^ Preschool: 2-4 years old; primary: 5-11 years old; secondary level: 12-18 years old.

^h^ Quality assessment was performed by review of study design, assessment of exposure, assessment of outcome, control of confounding, and evidence of bias. Each of the 5 quality criteria was evaluated and scored on an integer scale (0 or 1, with 1 being better) and summed; quality scores from 0 to 3 were considered lower quality and 4 to 5 higher quality.

^i^ Overall is reported when all studies and estimates have the same characteristic regarding the specific heterogeneity source explored.

CI, Confidence Intervals; RCT, randomized controlled trial; QED, quasi-experimental intervention; US=United States

# Table D. Prespecified sources of heterogeneity explored among interventions evaluating the effect of school meal standards on dietary intakes or meal contents in children.

| **Heterogeneity**  **sources ^a^** | **Total fat in-school lunch intake %E/d** | | **Total fat in-school content g/lunch** | | **Saturated fat in-school lunch intake %E/d** | | | **Saturated fat lunch content g/lunch** | | | **Total caloric in-school lunch intake kcal/d** | | **Total caloric lunch content kcal/lunch** | | **Sodium in-school lunch intake mg/d** | |
| --- | --- | --- | --- | --- | --- | --- | --- | --- | --- | --- | --- | --- | --- | --- | --- | --- |
|  | N (n)^b^ | Mean (95% CI)^c^ | N (n)^b^ | Mean (95% CI)^c^ | N (n)^b^ | | Mean (95% CI)^c^ | N (n)^b^ | | Mean (95% CI)^c^ | N (n)^b^ | Mean (95% CI)^c^ | N (n)^b^ | Mean (95% CI)^c^ | N (n)^b^ | Mean (95% CI)^c^ |
| **Overall** | 9 (9) | -8.27 (-10.03, -6.52) | 10 (11) | -3.35 (-5.20, -1.50) | 9 (9) | -2.75 (-4.39, -1.11) | | 7 (8) | -1.27 (-1.85, -0.68) | | 11 (11) | -28 (-76, 20) | 9 (10) | -56 (-76, -36) | 6 (6) | -227 (-384, -69) |
| **Study design** |  |  |  |  |  |  | |  |  | |  |  |  |  |  |  |
| RCT | 2 (2) | -7.15 (-11.36, -2.95) | 3 (3) | -3.94 (-6.38, -1.49) | 2 (2) | -2.74 (-4.99, -0.48) | | 2 (2) | -1.79 (-2.88, -0.70) | | 2 (2) | -58 (-84, -33) | 3 (3) | -37 (-71,-4) | 1 (1) | n/a) |
| QED | 7 (7) | -8.78 (-10.79, -6.76) | 7 (8) | -3.18 (-5.65, -0.72) | 7 (7) | -2.77 (-4.82, -0.73) | | 5 (6) | -0.89 (-1.31, -0.48) | | 9 (9) | -23 (-80, 34) | 6 (7) | -68 (-91, -44) | 5 (5) | -268 (-456, -79) |
| **Region ^d^** |  |  |  |  |  |  | |  |  | |  |  |  |  |  |  |
| US/Canada | 5 (5) | -6.91 (-8.89, -4.94) ^j^ | 10 (11) | Overall ^k^ | 5 (5) | -2.66 (-4.88, -0.44) | | 7 (8) | Overall ^k^ | | 7 (7) | 5 (-34, 45) | 9 (10) | Overall ^k^ | 2 (2) | -193 (-523, 137) |
| Europe/New Zealand | 4 (4) | -10.57 (-11.68, -9.47) ^j^ | 0 (0) | n/a | 4 (4) | -2.89 (-5.07, -0.70) | | 0 (0) | n/a | | 4 (4) | -87 (-250, 77) | 0 (0) | n/a | 4 (4) | -245 (-455, -36) |
| **Type of intervention ^e^** |  |  |  |  |  |  | |  |  | |  |  |  |  |  |  |
| Food policy only | 5 (5) | -10.32 (-11.69, -8.95) | 3 (3) | -1.94 (-5.49, 1.60) | 5 (5) | -2.28 (-4.76, 0.20) | | 3 (3) | -1.07 (-1.67, -0.48) | | 6 (6) | -48 (-123, 27) | 2 (2) | -37 (-121, 47) | 4 (4) | -245 (-455, -36) |
| Multi-component | 4 (4) | -7.05 (-9.10, -4.99) | 7 (8) | -3.88 (-6.08, -1.68) | 4 (4) | -3.40 (-4.45, -2.35) | | 4 (5) | -1.33 (-2.18, -0.48) | | 5 (5) | -1 (-64, 62) | 7 (8) | -60 (-80, -40) | 2 (2) | -193 (-523, 137) |
| **Non-dietary targets ^f^** |  |  |  |  |  |  | |  |  | |  |  |  |  |  |  |
| No | 6 (6) | -9.08 (-11.13, -7.02) | 6 (7) | -2.13 (-4.17, -0.09) | 6 (6) | -2.61 (-4.80, -0.42) | | 5 (6) | -0.89 (-1.31, -0.48) | | 8 (8) | -24 (-83, 36) | 5 (6) | -63 (-94, -33) | 4 (4) | -245 (-455, -36) |
| Yes | 3 (3) | -6.77 (-10.31, -3.23) | 4 (4) | -4.68 (-6.64, -2.73) | 3 (3) | -3.02 (-4.78, -1.25) | | 2 (2) | -1.79 (-2.88, -0.70) | | 3 (3) | -57 (-82, -32) | 4 (4) | -48 (-77, -19) | 2 (2) | -193 (-523, 137) |
| **Νο of environmental strategies ^g^** | | |  |  |  |  | |  |  | |  |  |  |  |  |  |
| 1 | 8 (8) | -8.42 (-10.19, -6.65) | 9 (10) | -3.47 (-5.58, -1.36) | 8 (8) | -3.16 (-4.23, -2.09) | | 7 (8) | Overall ^k^ | | 10 (10) | -39 (-90, 11) | 8 (9) | -59 (-80, -37) | 6 (6) | Overall ^k^ |
| >1 | 1 (1) | n/a | 1 (1) | n/a | 1 (1) | n/a | | 0 (0) | n/a | | 1 (1) | n/a | 1 (1) | n/a | 0 (0) | n/a |
| **School level ^h^** |  |  |  |  |  |  | |  |  | |  |  |  |  |  |  |
| Preschool | 1 (1) | n/a | 1 (1) | n/a | 1 (1) | n/a | | 1 (1) | n/a | | 1 (1) | n/a | 1 (1) | n/a |  |  |
| Primary | 5 (5) | -7.98 (-10.70, -5.25) | 6 (6) | -5.26 (-6.94, -3.58) | 5 (5) | -3.32 (-4.73, -1.91) | | 4 (4) | -1.56 (-2.40, -0.73) | | 6 (6) | -4 (-63, 55) | 5 (5) | -62 (-92, -33) | 4 (4) | -134 (-238, -29) |
| Secondary | 3 (3) | -9.80 (-11.14, -8.45) | 2 (2) | -0.78 (-4.97, 3.41) | 3 (3) | -1.43 (-3.01, 0.14) | | 1 (1) | n/a | | 3 (3) | -95 (-288, 99) | 2 (2) | -25 (-62, 12) | 2 (2) | -385 (-444, -325) |
| Primary and secondary |  |  | 1 (2) | -0.83 (-1.90, 0.23) |  |  | | 1 (2) | -0.68 (-1.07, -0.29) | | 1 (1) | n/a | 1 (2) | -76 (-96, -57) |  |  |
| **Quality score ^i^** |  |  |  |  |  |  | |  |  | |  |  |  |  |  |  |
| Low | 5 (5) | -7.72 (-10.92, -4.52) | 5 (5) | -5.75 (-8.51, -3.00) | 5 (5) | -3.00 (-5.86, -0.14) | | 3 (3) | -1.68 (-3.09, -0.27) | | 6 (6) | 44 (29, 58) ^j^ | 4 (4) | -74 (-109, -40) | 3 (3) | -189 (-359, -20) |
| High | 4 (4) | -8.43 (-10.68, -6.18) | 5 (6) | -1.98 (-4.31, 0.35) | 4 (4) | -2.51 (-3.75, -1.26) | | 4 (5) | -1.18 (-1.90, -0.46) | | 5 (5) | -99 (-174, -25) ^j^ | 5 (6) | -49 (-74, -24) | 3 (3) | -253 (-504, -3) |

^a^ Results are presented for selected heterogeneity sources (common across the three strategies of school food environment identified (Table 2, Table C in S1 File) for the outcomes with the largest numbers of estimates. For all other outcomes not presented, no significant heterogeneity sources were identified.

^b^ Number of estimates (values in parentheses) can be higher than number of studies included in the meta-analyses if multiple intervention groups or multiple comparisons were available from the same study.

^c^ Study-specific effect sizes were pooled using stratified inverse-variance weighted random-effect models (metan command in Stata). Effect sizes correspond to mean changes standardized across studies to consistent units; and precision estimates to 95% confidence intervals (CIs).

^d^ All studies in the category US/Canada refer to US studies and all studies in the category Europe/New Zealand refer to United Kingdom studies.

^e^ Single-component interventions consisted only of the school food environment policy. Multi-component interventions were included only if the food environment policy was a major component, judged qualitatively to be at least 30% of the overall intervention. Additional potential components included education, food/menu labeling, etc. (see Table 1).

^f^ In addition to the dietary targets, specific interventions also targeted non-dietary targets, such as physical activity and smoking.

^g^ School food environment policy strategies included direct provision of healthful foods, quality standards for competitive foods/ beverages, and quality standards for school meals.

^h^ Preschool: 2-4 years old; primary: 5-11 years old; secondary level: 12-18 years old.

^i^ Quality assessment was performed by review of study design, assessment of exposure, assessment of outcome, control of confounding, and evidence of bias. Each of the 5 quality criteria was evaluated and scored on an integer scale (0 or 1, with 1 being better) and summed; quality scores from 0 to 3 were considered lower quality and 4 to 5 higher quality.

^j^ Statistically significant (P<0.05) in univariate meta-regression analysis.

^k^ Overall is reported when all studies and estimates have the same characteristic regarding the specific heterogeneity source explored.

CI, Confidence Intervals; RCT, randomized controlled trial; QED, quasi-experimental intervention; US=United States


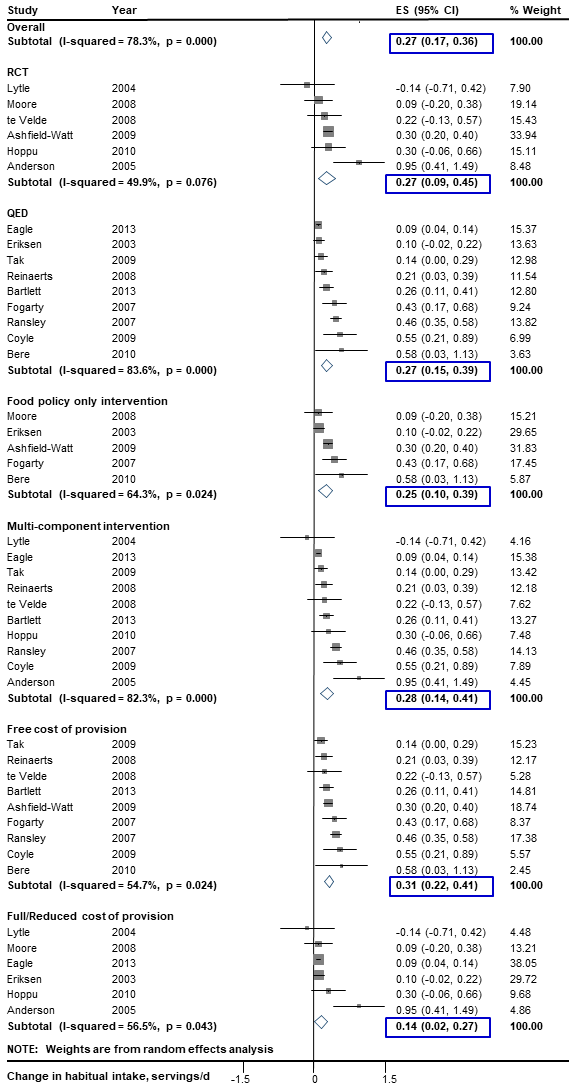
Figure A. Effect of direct provision of fruits and vegetables in schools on fruit intake in children by prespecified sources of heterogeneity. Solid squares represent study specific changes in prevalence of overweight (≥85th to <95th percentile), obesity (≥95th percentile) or overweigh/obesity combined (≥85th percentile); and lines, 95% confidence intervals (CIs). Vertical line represents pooled effect size (ES); and open diamond, corresponding 95% CI. Multi-component strategies were included only if the food environment policy was a major component, judged qualitatively to be at least 30% of the overall intervention. The relative contribution of the food environment policy component to the overall intervention was qualitatively assessed as low (30 to <60%), medium (60 to <90%), and high (≥90%).

RCT, randomized controlled trial; QED, quasi-experimental design.


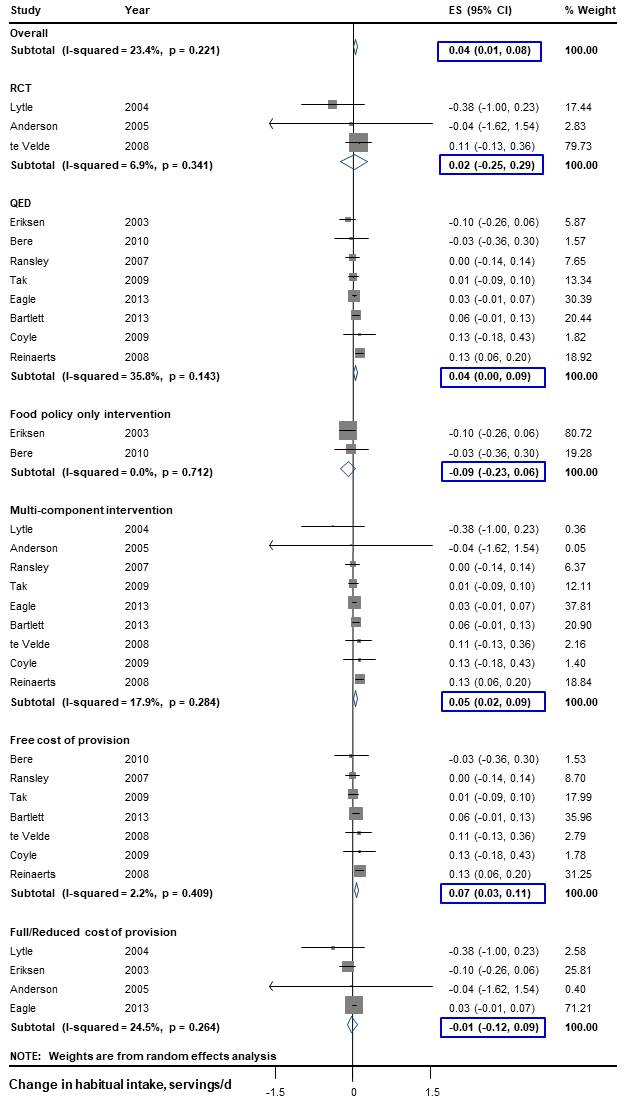


Figure B. Effect of direct provision of fruits and vegetables in schools on vegetable intake in children by prespecified sources of heterogeneity. Solid squares represent study specific changes in prevalence of overweight (≥85th to <95th percentile), obesity (≥95th percentile) or overweigh/obesity combined (≥85th percentile); and lines, 95% confidence intervals (CIs). Vertical line represents pooled effect size (ES); and open diamond, corresponding 95% CI. Multi-component strategies were included only if the food environment policy was a major component, judged qualitatively to be at least 30% of the overall intervention. The relative contribution of the food environment policy component to the overall intervention was qualitatively assessed as low (30 to <60%), medium (60 to <90%), and high (≥90%).

RCT, randomized controlled trial; QED, quasi-experimental design.

**
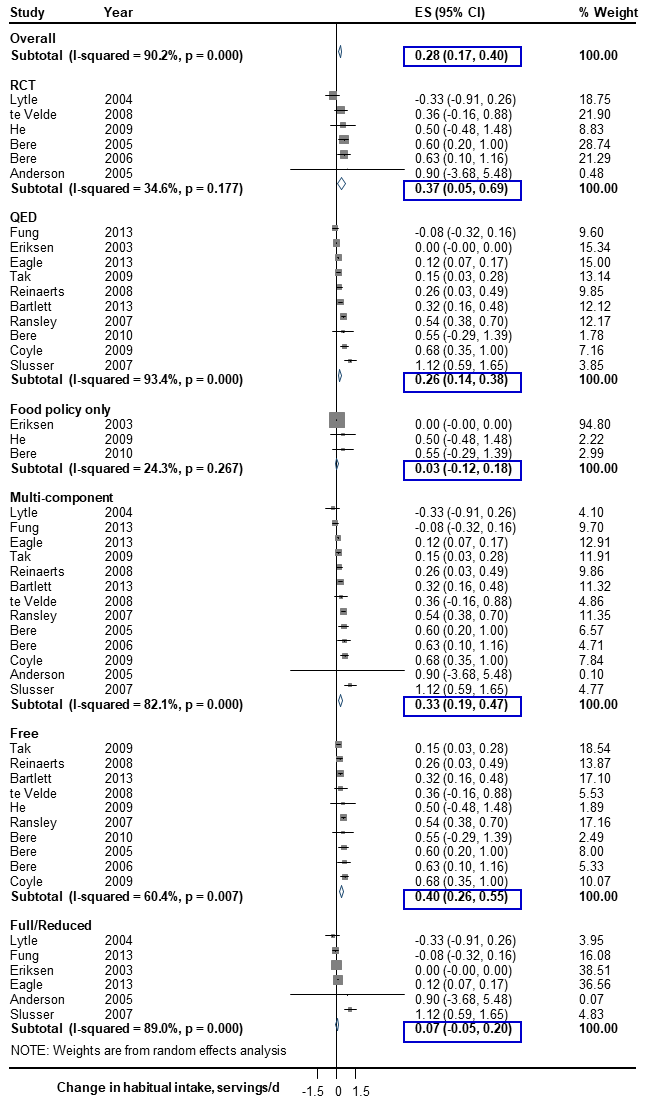
**

Figure C. Effect of direct provision of fruits and vegetables in schools on fruit and vegetable intake in children by prespecified sources of heterogeneity. Solid squares represent study specific changes in prevalence of overweight (≥85th to <95th percentile), obesity (≥95th percentile) or overweigh/obesity combined (≥85th percentile); and lines, 95% confidence intervals (CIs). Vertical line represents pooled effect size (ES); and open diamond, corresponding 95% CI. Multi-component strategies were included only if the food environment policy was a major component, judged qualitatively to be at least 30% of the overall intervention. The relative contribution of the food environment policy component to the overall intervention was qualitatively assessed as low (30 to <60%), medium (60 to <90%), and high (≥90%).

RCT, randomized controlled trial; QED, quasi-experimental design.


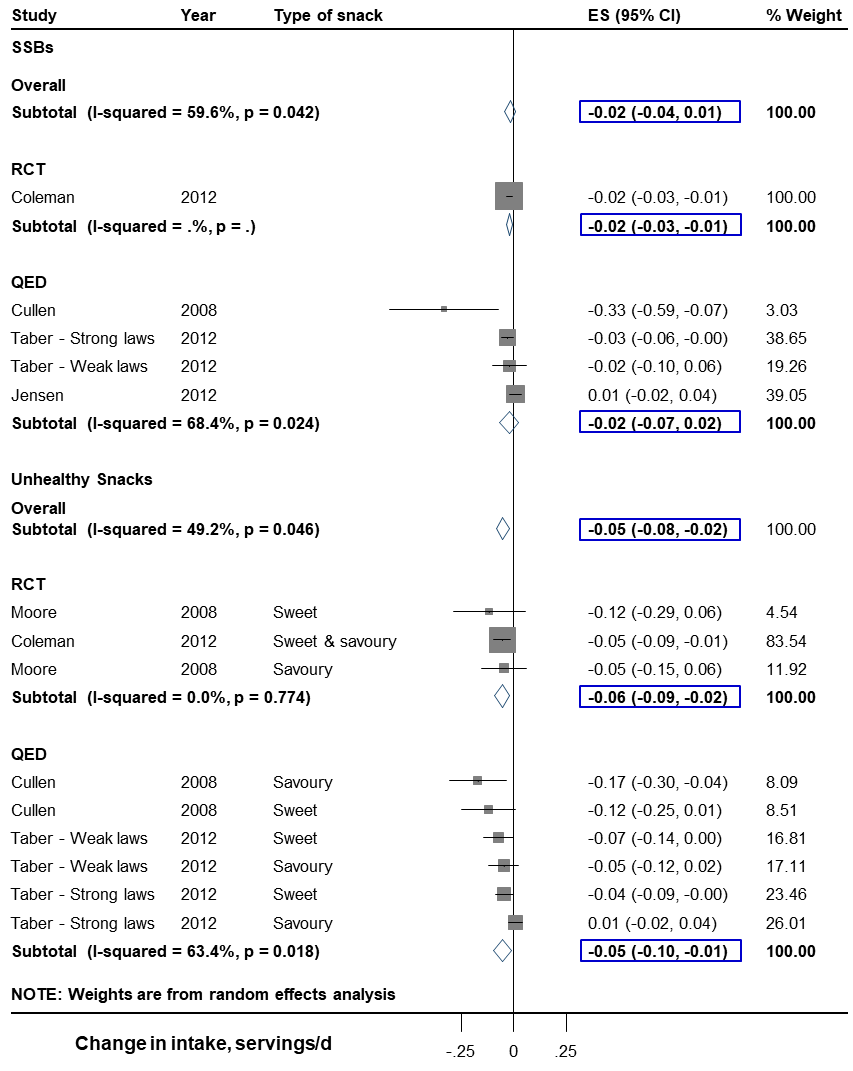


Figure D. Effect of competitive food and beverage standards in schools on sugar-sweetened beverages and unhealthy snack intake in children by prespecified sources of heterogeneity. Solid squares represent study specific changes in prevalence of overweight (≥85th to <95th percentile), obesity (≥95th percentile) or overweigh/obesity combined (≥85th percentile); and lines, 95% confidence intervals (CIs). Vertical line represents pooled effect size (ES); and open diamond, corresponding 95% CI.

RCT, randomized controlled trial; QED, quasi-experimental design.


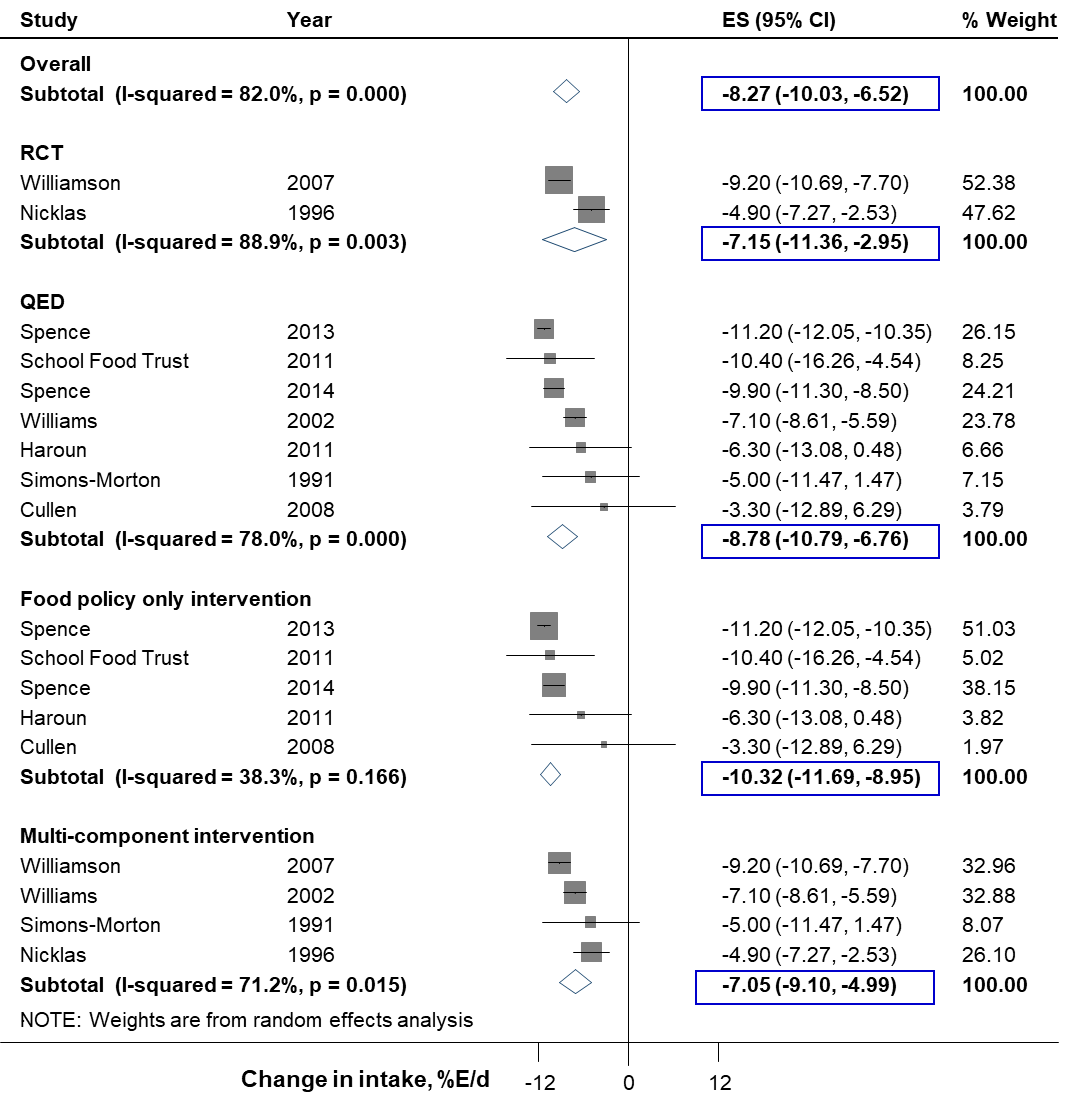


Figure E. Effect of school meal standards in schools on total fat intake in children by prespecified sources of heterogeneity. Solid squares represent study specific changes in prevalence of overweight (≥85th to <95th percentile), obesity (≥95th percentile) or overweigh/obesity combined (≥85th percentile); and lines, 95% confidence intervals (CIs). Vertical line represents pooled effect size (ES); and open diamond, corresponding 95% CI. Multi-component strategies were included only if the food environment policy was a major component, judged qualitatively to be at least 30% of the overall intervention. The relative contribution of the food environment policy component to the overall intervention was qualitatively assessed as low (30 to <60%), medium (60 to <90%), and high (≥90%).

RCT, randomized controlled trial; QED, quasi-experimental design.


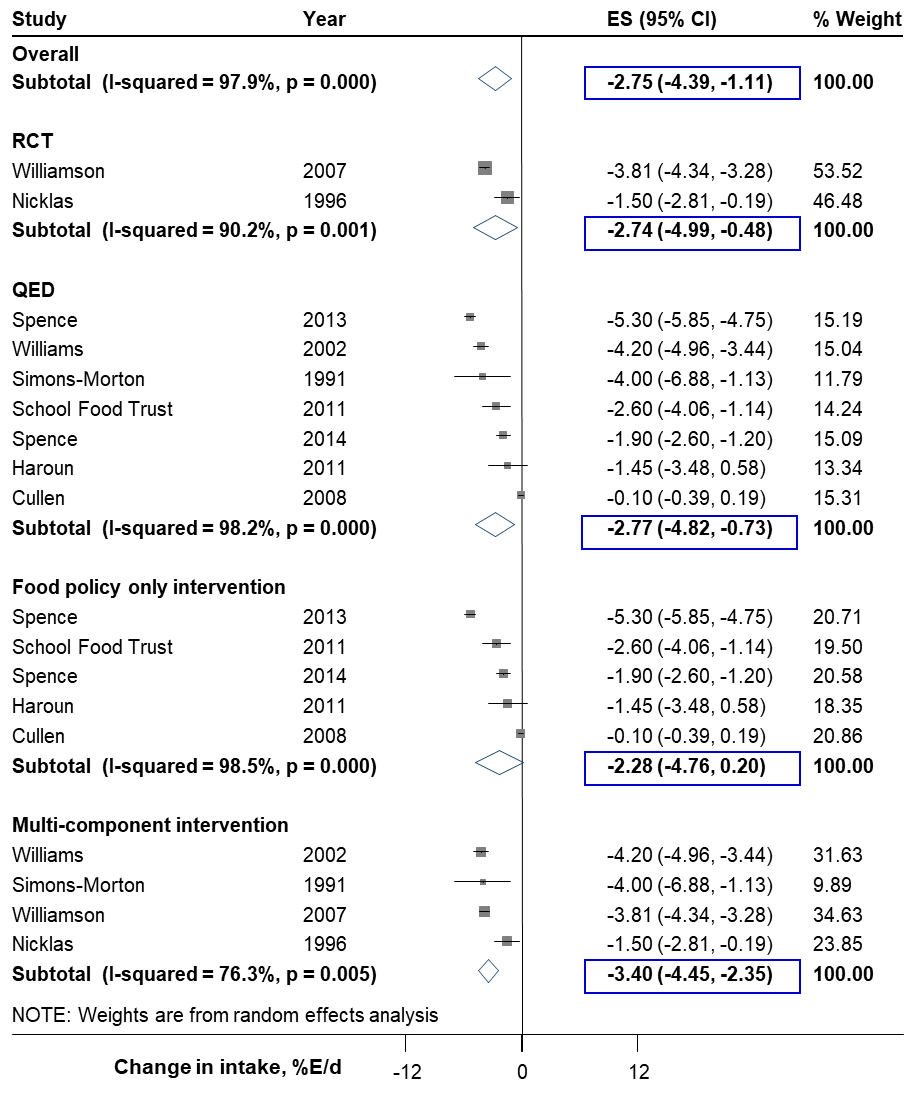


Figure F. Effect of school meal standards in schools on saturated fat intake in children by prespecified sources of heterogeneity. Solid squares represent study specific changes in prevalence of overweight (≥85th to <95th percentile), obesity (≥95th percentile) or overweigh/obesity combined (≥85th percentile); and lines, 95% confidence intervals (CIs). Vertical line represents pooled effect size (ES); and open diamond, corresponding 95% CI. Multi-component strategies were included only if the food environment policy was a major component, judged qualitatively to be at least 30% of the overall intervention. The relative contribution of the food environment policy component to the overall intervention was qualitatively assessed as low (30 to <60%), medium (60 to <90%), and high (≥90%).

RCT, randomized controlled trial; QED, quasi-experimental design.


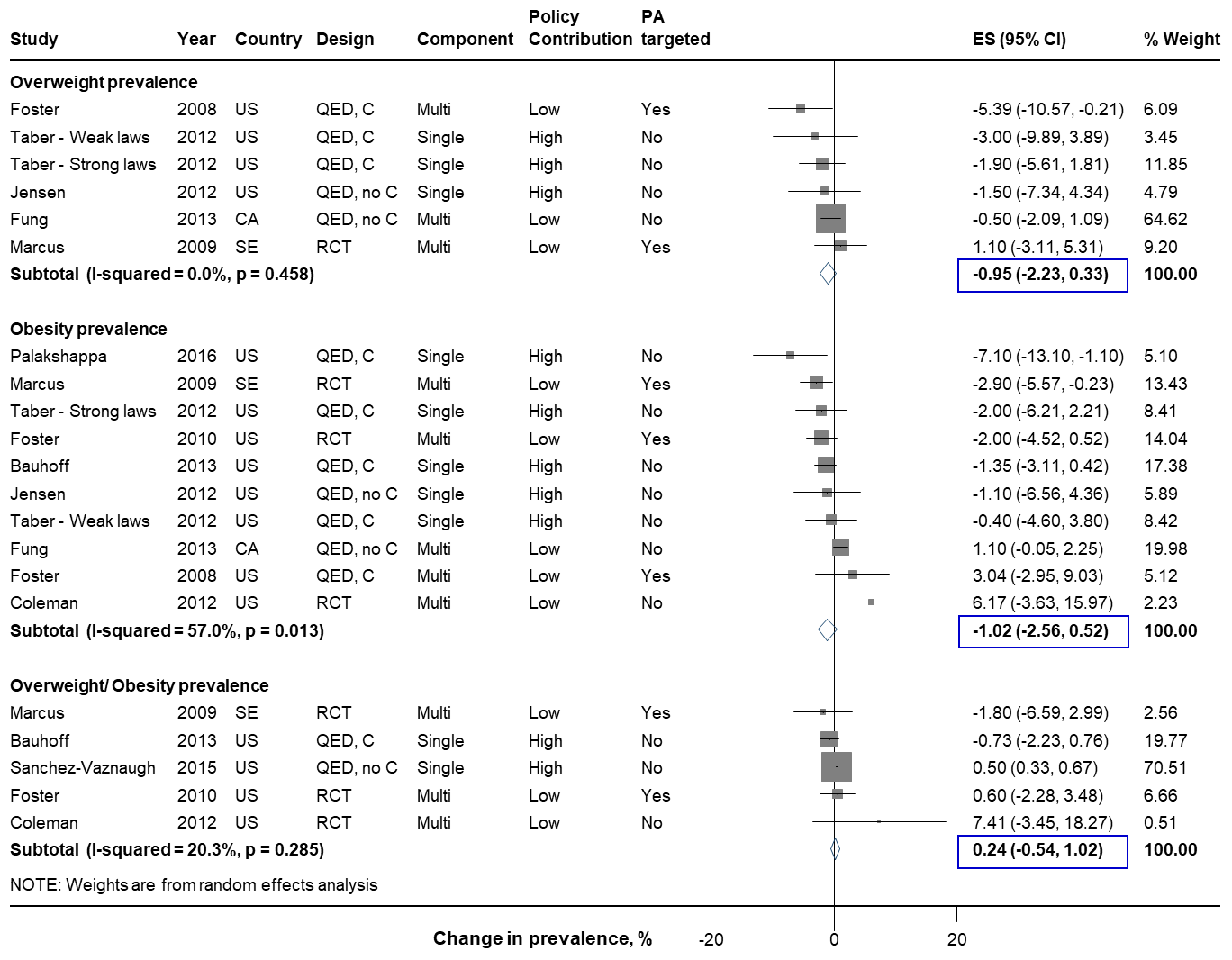


Figure G. Effect of competitive food and beverage standards in schools on overweight and obesity prevalence in children. Solid squares represent study specific changes in prevalence of overweight (≥85th to <95th percentile), obesity (≥95th percentile) or overweigh/obesity combined (≥85th percentile); and lines, 95% confidence intervals (CIs). Vertical line represents pooled effect size (ES); and open diamond, corresponding 95% CI. Multi-component strategies were included only if the food environment policy was a major component, judged qualitatively to be at least 30% of the overall intervention. The relative contribution of the food environment policy component to the overall intervention was qualitatively assessed as low (30 to <60%), medium (60 to <90%), and high (≥90%).

BMI, body mass index; PA, physical activity; RCT, randomized controlled trial; QED, quasi-experimental intervention with external control group; QED, no C, quasi-experimental intervention without external control group; CA, Canada; SE, Sweden; US, United States of America.


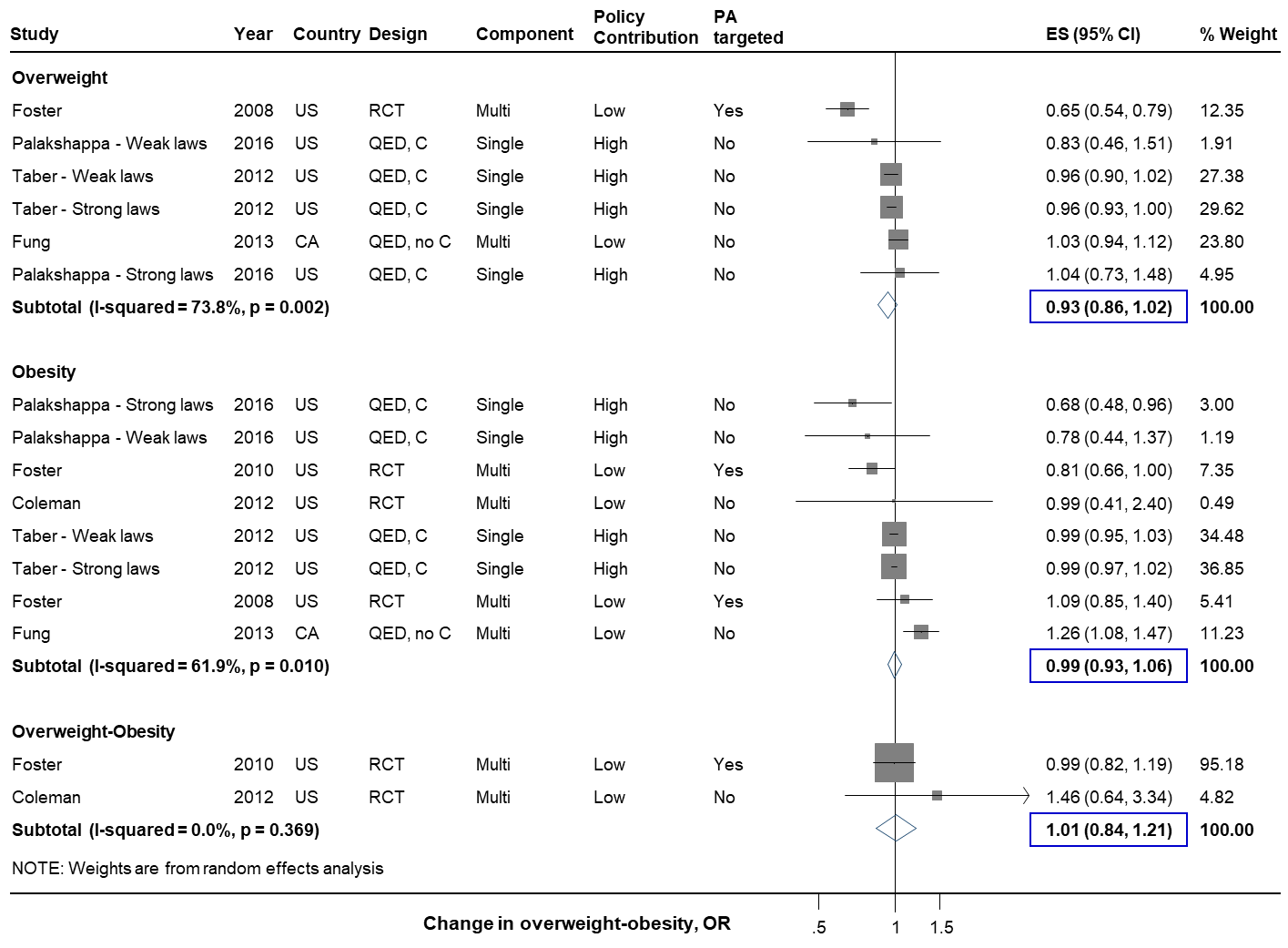


Figure H. Effect of competitive food and beverage standards in schools on odds of overweight and obesity in children. Solid squares represent study specific changes in odds of overweight (≥85th to <95th percentile), obesity (≥95th percentile) or overweigh/obesity combined (≥85th percentile); and lines, 95% confidence intervals (CIs). Vertical line represents pooled effect size (ES); and open diamond, corresponding 95% CI. Multi-component strategies were included only if food environment policy was a major component, judged qualitatively to be at least 30% of the overall intervention. The relative contribution of the food environment policy component to the overall intervention was qualitatively assessed as low (30 to <60%), medium (60 to <90%), and high (≥90%).

BMI, body mass index; PA, physical activity; RCT, randomized controlled trial; QED, quasi-experimental intervention with external control group; QED, no C, quasi-experimental intervention without external control group; CA, Canada; US, United States of America.


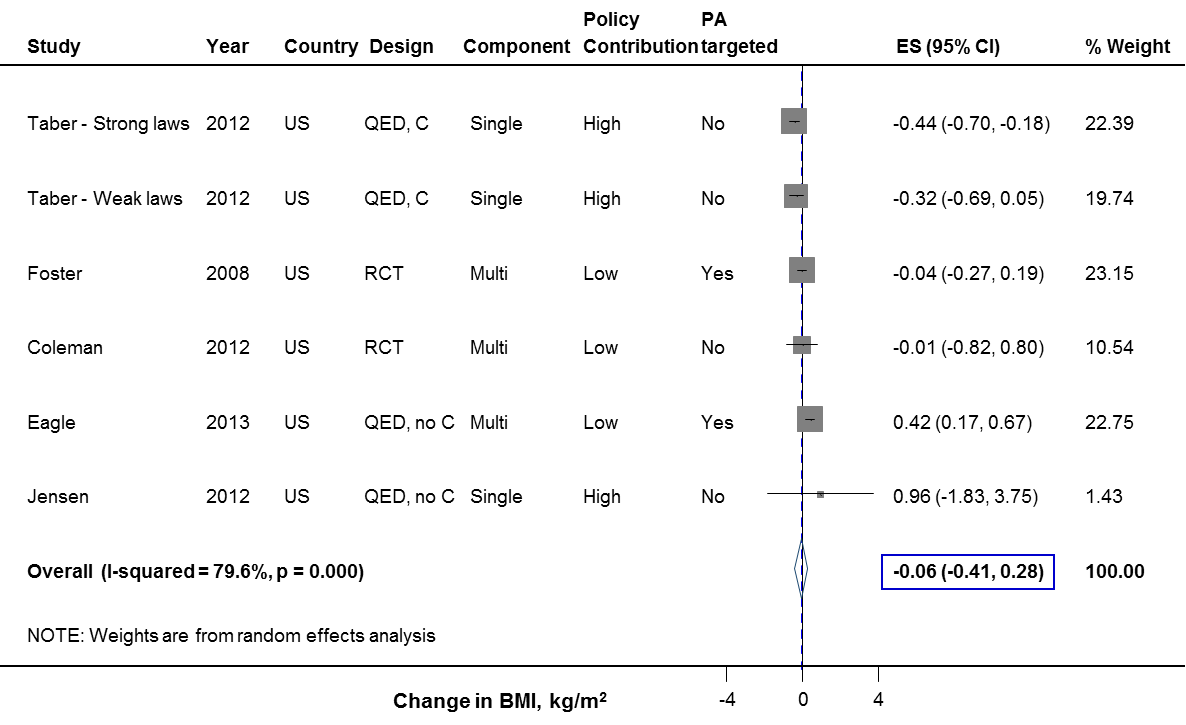


Figure I. Effect of competitive food and beverage standards in schools on BMI in children. Solid squares represent study specific continuous changes in body mass index (BMI, kg/m^2^); and lines, 95% confidence intervals (CIs). Vertical line represents pooled effect size (ES); and open diamond, corresponding 95% CI. Multi-component strategies were included only if food environment policy was a major component, judged qualitatively to be at least 30% of the overall intervention. The relative contribution of the food environment policy component to the overall intervention was qualitatively assessed as low (30 to <60%), medium (60 to <90%), and high (≥90%).

PA, physical activity; RCT, randomized controlled trial; QED, quasi-experimental intervention with external control group; QED, no C, quasi-experimental intervention without external control group; US, United States of America.


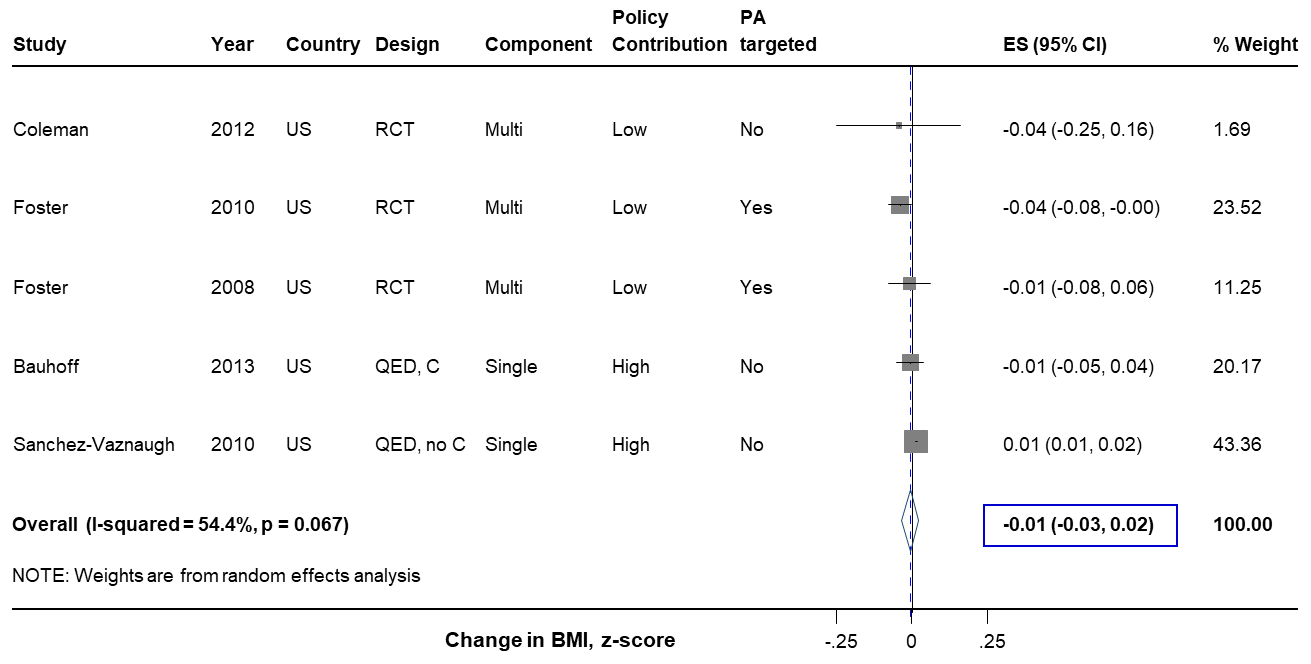


Figure J. Effect of competitive food and beverage standards in schools on BMI z-score in children. Solid squares represent study specific continuous changes in body mass index (BMI, z-score); and lines, 95% confidence intervals (CIs). Vertical line represents pooled effect size (ES); and open diamond, corresponding 95% CI. Multi-component strategies were included only if food environment policy was a major component, judged qualitatively to be at least 30% of the overall intervention. The relative contribution of the food environment policy component to the overall intervention was qualitatively assessed as low (30 to <60%), medium (60 to <90%), and high (≥90%).

PA, physical activity; RCT, randomized controlled trial; QED, quasi-experimental intervention with external control group; QED, no C, quasi-experimental intervention without external control group; US, United States of America.


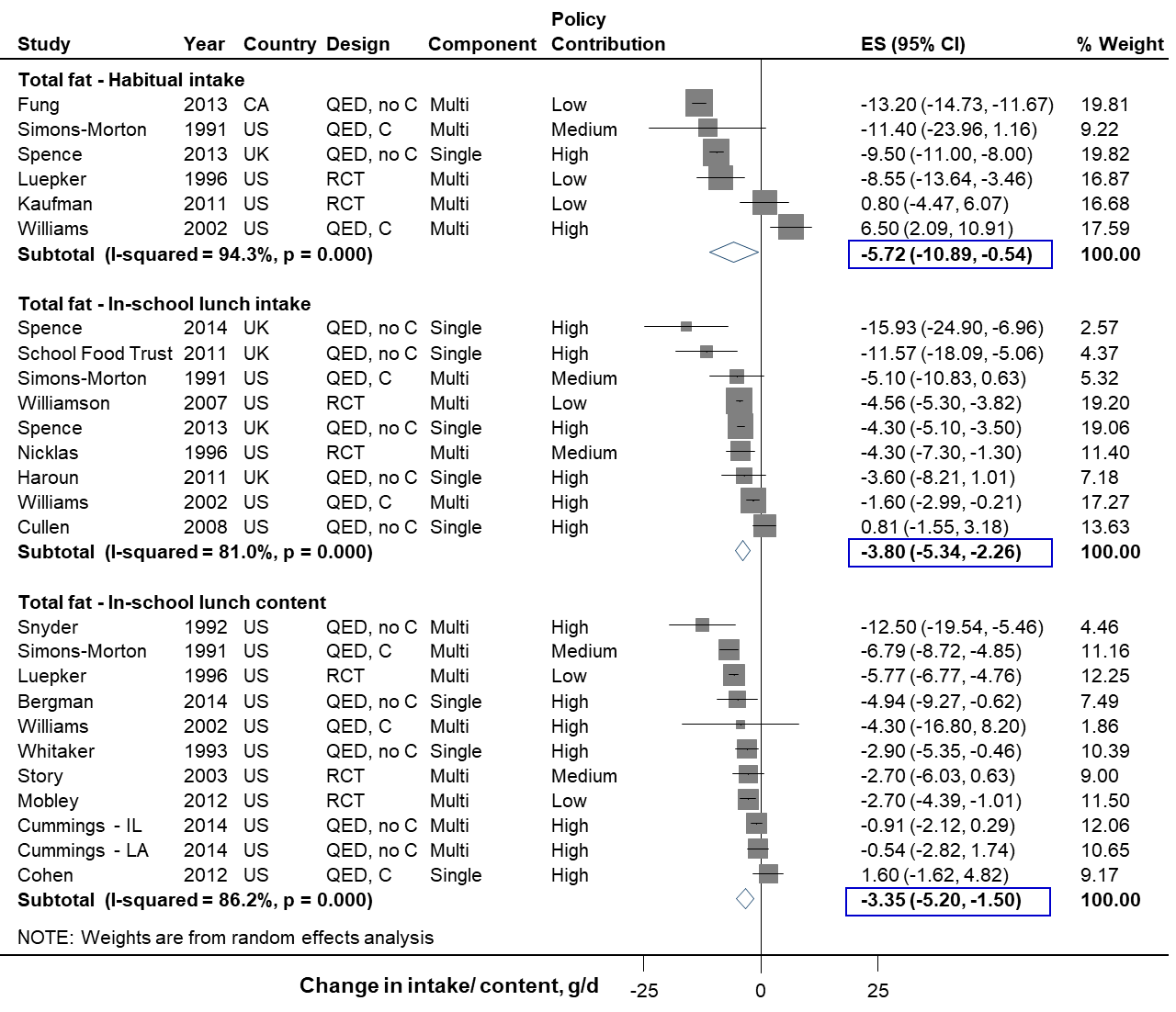


Figure K. Effect of school meal standards on total fat intake or meal content in children. Intakes represent total daily or in-school lunch consumption, and contents in-school lunch content. Solid squares represent study specific continuous changes in reported intakes or contents; and lines, 95% confidence intervals (CIs). Vertical line represents pooled effect size (ES); and open diamond, corresponding 95% CI. Multi-component strategies were included only if the food environment policy was a major component, judged qualitatively to be at least 30% of the overall intervention. The relative contribution of the food environment policy component to the overall intervention was qualitatively assessed as low (30 to <60%), medium (60 to <90%), and high (≥90%). In secondary analysis, in-school meal (lunch or breakfast) total fat consumption decreased by 3.71 g/d (N=10; -5.20, -2.22), and in-school meal total fat content decreased by 2.97 g/meal (N=15; -4.78, -1.16).

RCT, randomized controlled trial; QED, quasi-experimental intervention with external control group; QED, no C, quasi-experimental intervention without external control group; UK, United Kingdom; US, United States of America.


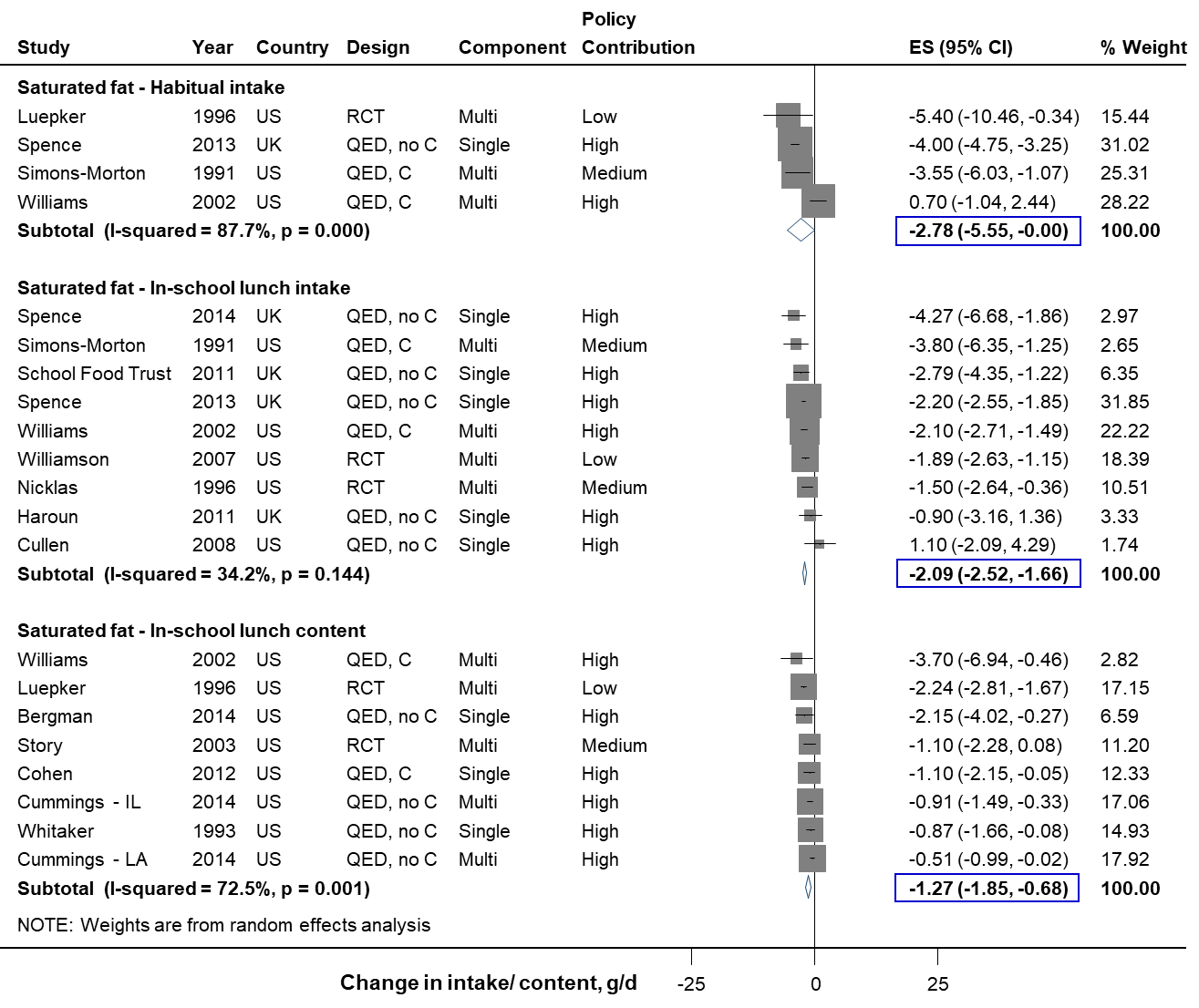


Figure L. Effect of school meal standards on saturated fat intake or meal content in children. Intakes represent total daily or in-school lunch consumption, and contents in-school lunch content. Solid squares represent study specific continuous changes in reported intakes; and lines, 95% confidence intervals (CIs). Vertical line represents pooled effect size (ES); and open diamond, corresponding 95% CI. Multi-component strategies were included only if the food environment policy was a major component, judged qualitatively to be at least 30% of the overall intervention. The relative contribution of the food environment policy component to the overall intervention was qualitatively assessed as low (30 to <60%), medium (60 to <90%), and high (≥90%). In secondary analysis, in-school meal (lunch or breakfast) saturated fat consumption decreased by 1.82 g/d (N=10; -2.53, -1.11), and in-school meal saturated fat content decreased by 1.27 g/meal (N=11; -1.94, -0.59).
RCT, randomized controlled trial; QED, quasi-experimental intervention with external control group; QED, no C, quasi-experimental intervention without external control group; UK, United Kingdom; US, United States of America.


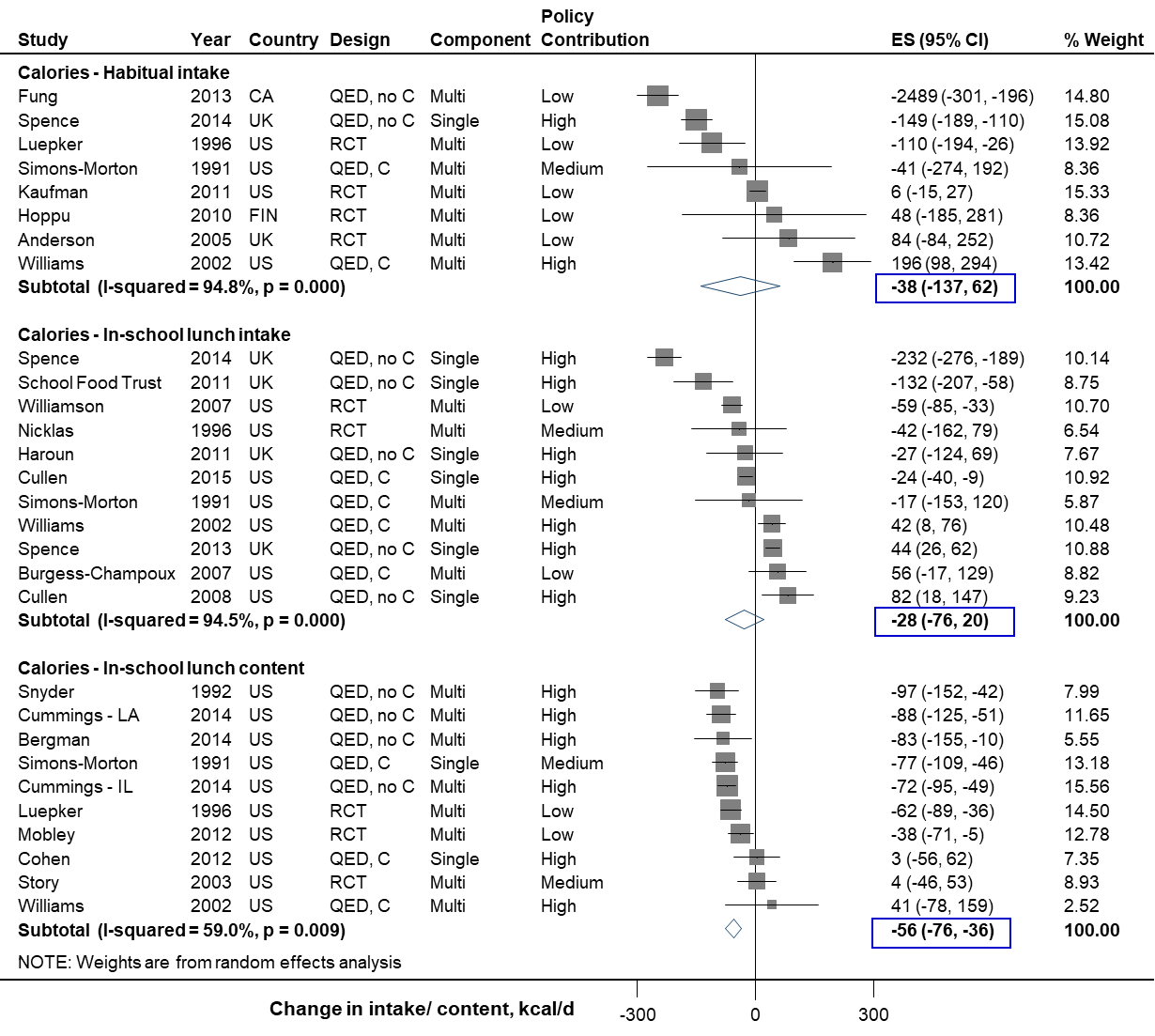


Figure M. Effect of school meal standards on total caloric intake or meal content in children. Intakes represent total daily or in-school lunch consumption, and contents in-school lunch content. Solid squares represent study specific continuous changes in reported intakes; and lines, 95% confidence intervals (CIs). Vertical line represents pooled effect size (ES); and open diamond, corresponding 95% CI. Multi-component strategies were included only if the food environment policy was a major component, judged qualitatively to be at least 30% of the overall intervention. The relative contribution of the food environment policy component to the overall intervention was qualitatively assessed as low (30 to <60%), medium (60 to <90%), and high (≥90%). In secondary analysis, in-school meal (lunch or breakfast) caloric consumption decreased by 29 kcal/d (N=12; -76, 18), and in-school meal caloric content decreased by 57 kcal/meal (N=14; -98, -16).
RCT, randomized controlled trial; QED, quasi-experimental intervention with external control group; QED, no C, quasi-experimental intervention without external control group; CA, Canada; FIN, Finland; UK, United Kingdom; US, United States of America.


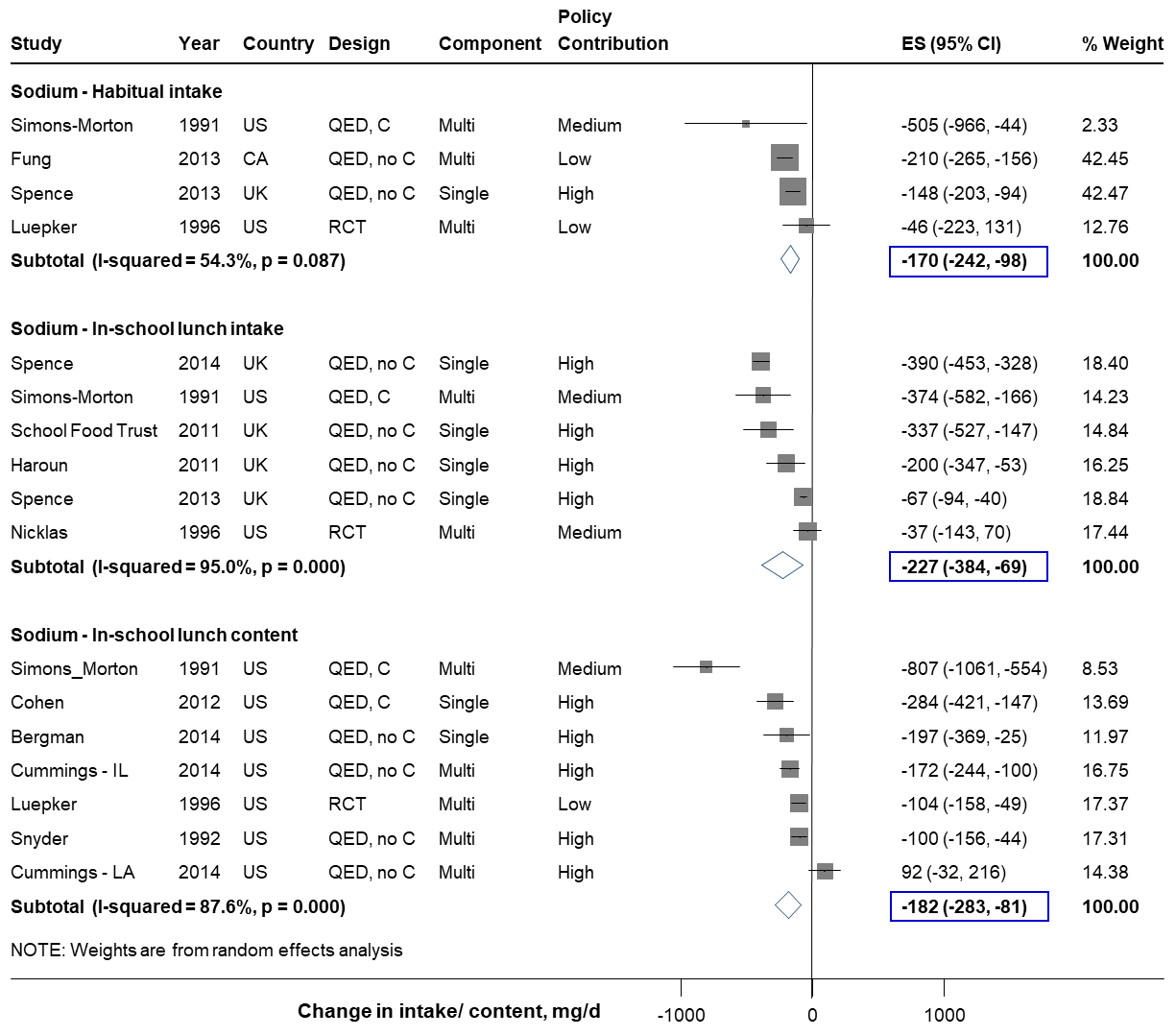


Figure N. Effect of school meal standards on sodium intake or meal content in children. Intakes represent total daily or in-school lunch consumption, and contents in-school lunch content. Solid squares represent study specific continuous changes in reported intakes; and lines, 95% confidence intervals (CIs). Vertical line represents pooled effect size (ES); and open diamond, corresponding 95% CI. Multi-component strategies were included only if the food environment policy was a major component, judged qualitatively to be at least 30% of the overall intervention. The relative contribution of the food environment policy component to the overall intervention was qualitatively assessed as low (30 to <60%), medium (60 to <90%), and high (≥90%). In secondary analysis, in-school meal (lunch or breakfast) sodium consumption decreased by 221 mg/d (N=7; -371, -71), and in-school meal sodium content decreased by 172 mg/meal (N=10; -287, -57).
RCT, randomized controlled trial; QED, quasi-experimental intervention with external control group; QED, no C, quasi-experimental intervention without external control group; CA, Canada; UK, United Kingdom; US, United States of America.

**Fruit intake** **Vegetable intake**


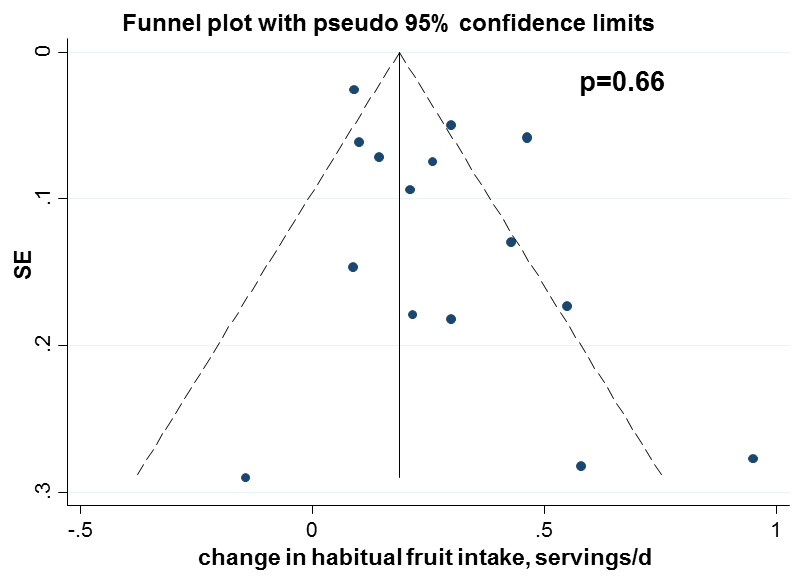

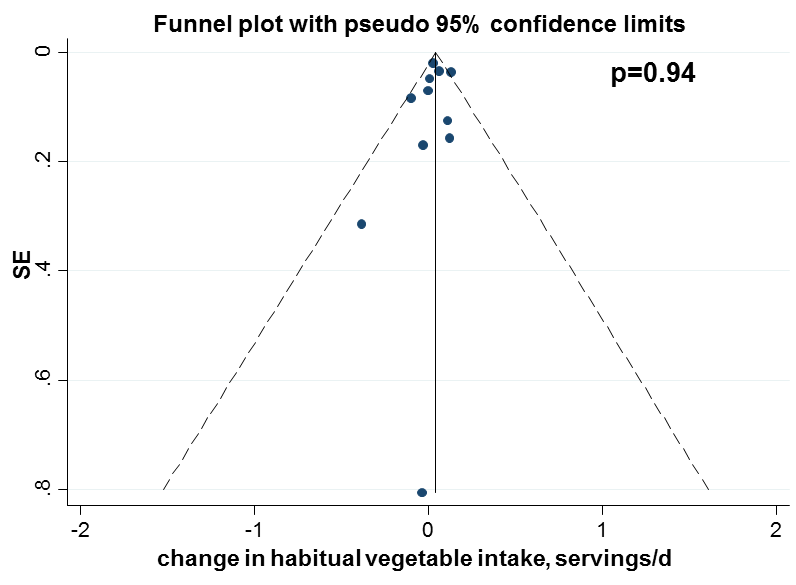


**Fruit and vegetable intake Caloric intake**


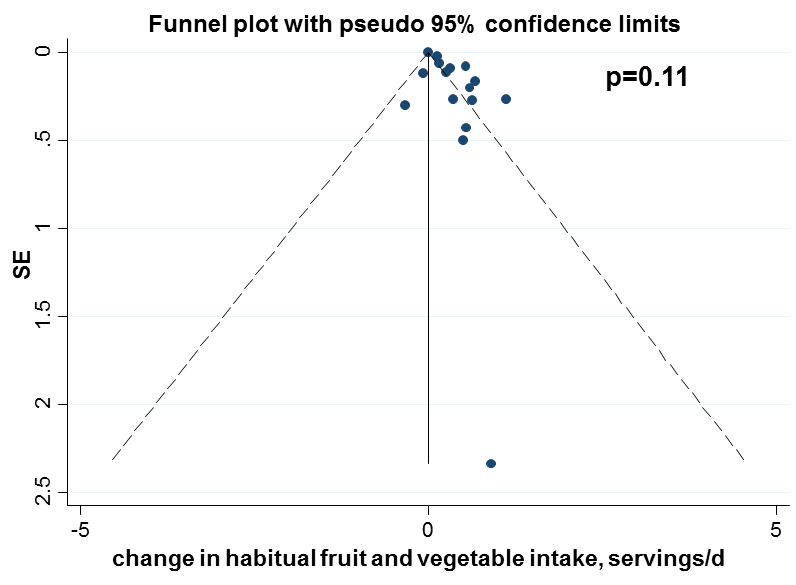

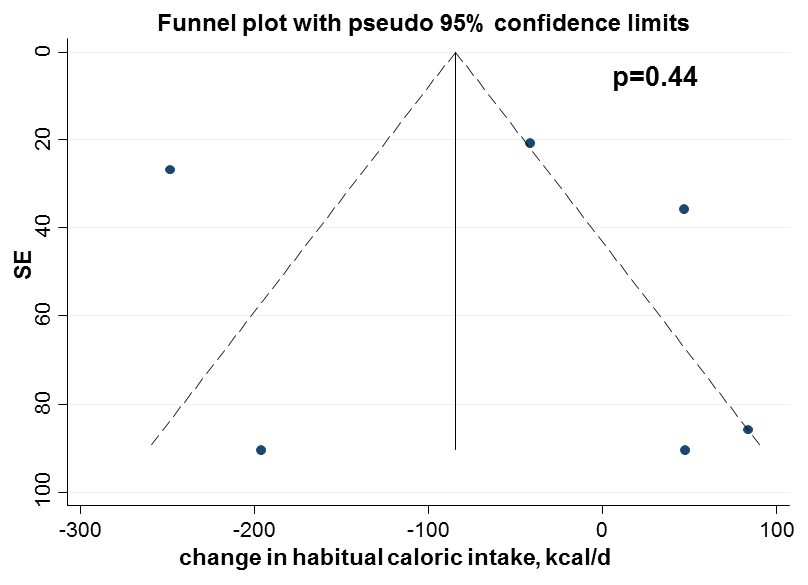


Figure O. Begg’s funnel plots for graphical evaluation of potential publication bias for the effect of direct provision of fruits and vegetables in schools on fruit, vegetable and caloric intake in children. Intakes represent total daily (not just in-school consumption). P-values based on the Begg’s adjusted rank-correlation test for presence of publication bias.

**SSB intake Unhealthy snack intake**


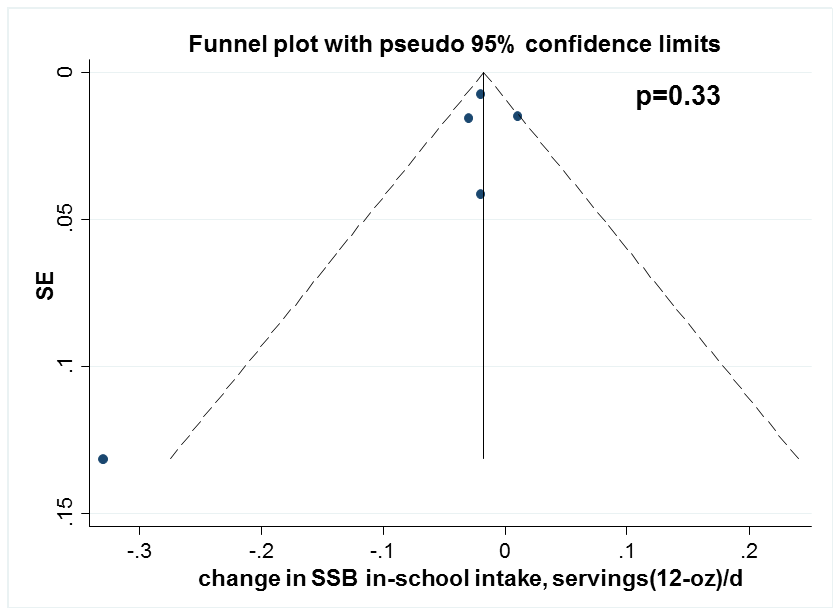

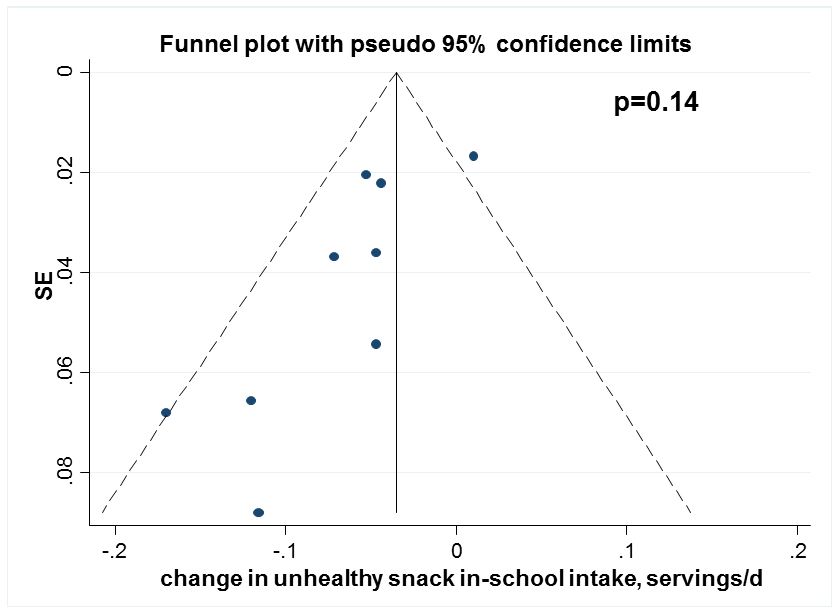


**Obesity prevalence BMI**


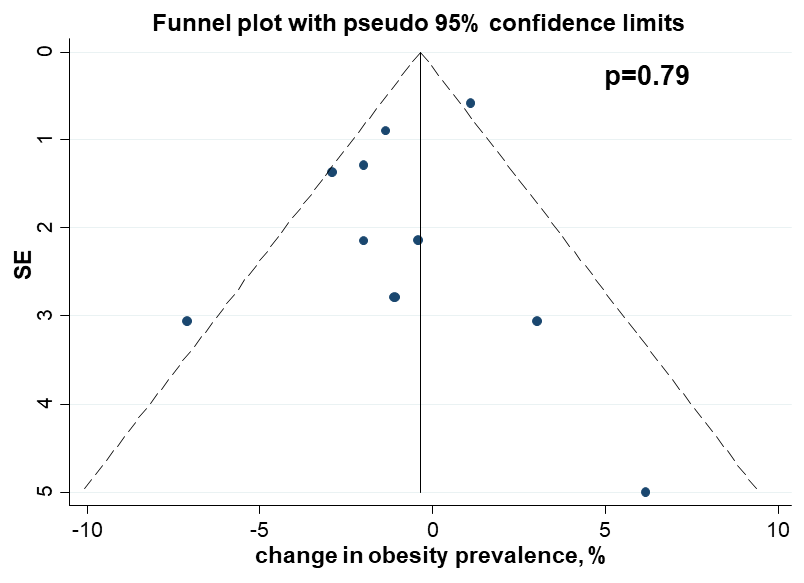

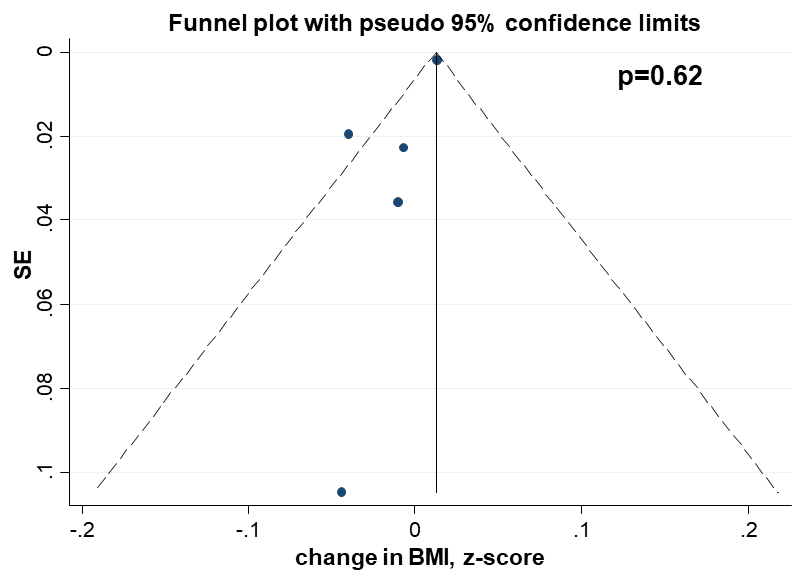


**Caloric intake**


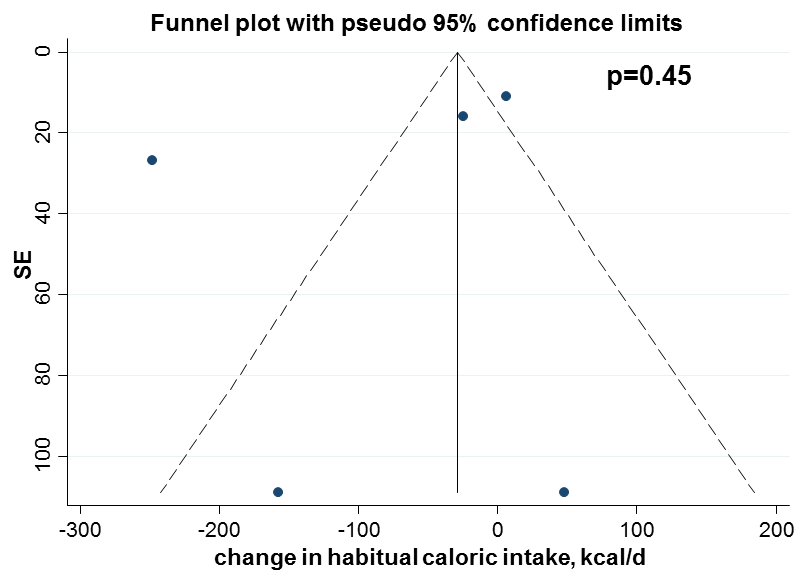


Figure P. Begg’s funnel plots for graphical evaluation of potential publication bias for the effect of competitive food and beverage standards in schools on dietary intakes or adiposity in children. Intakes represent total in-school consumption. P-values based on the Begg’s adjusted rank-correlation test for presence of publication bias.

**Total fat intake Saturated fat intake**


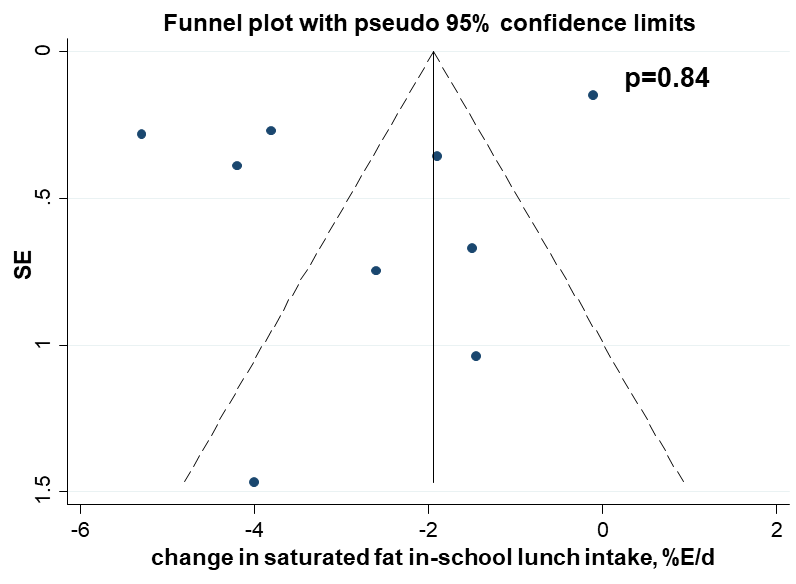

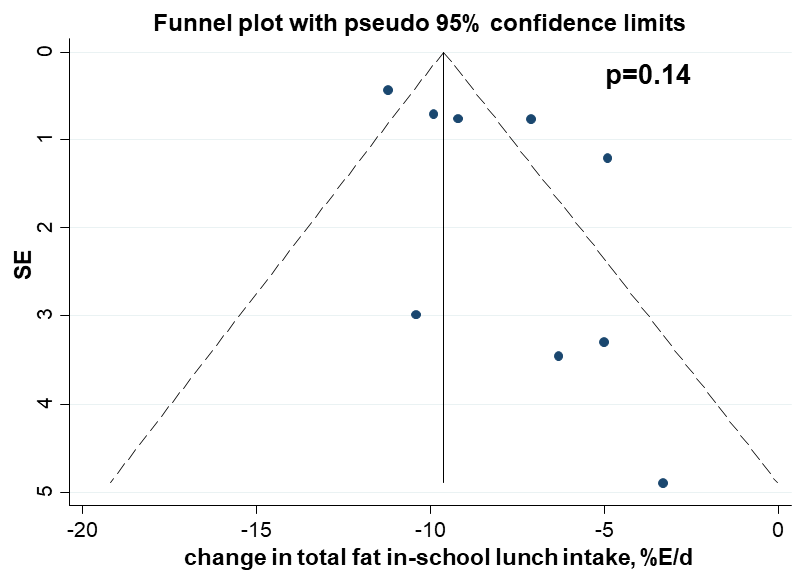


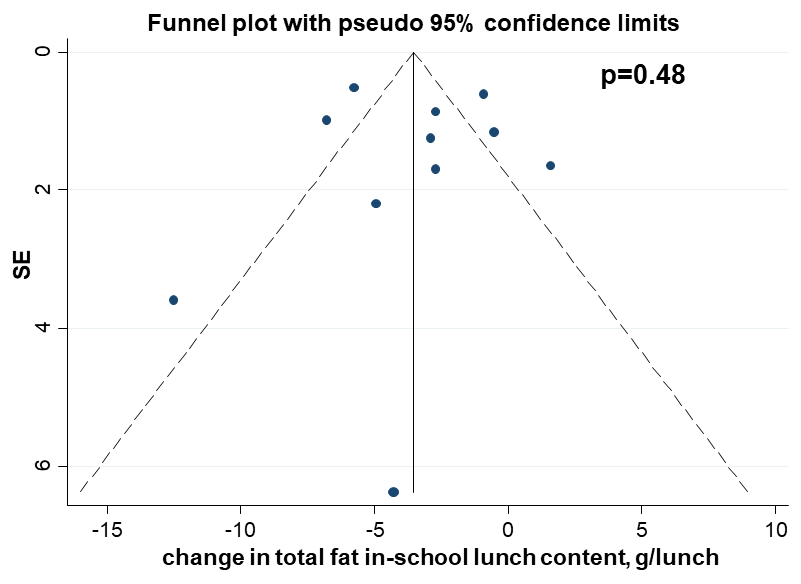
**Total fat lunch content Saturated fat lunch content**


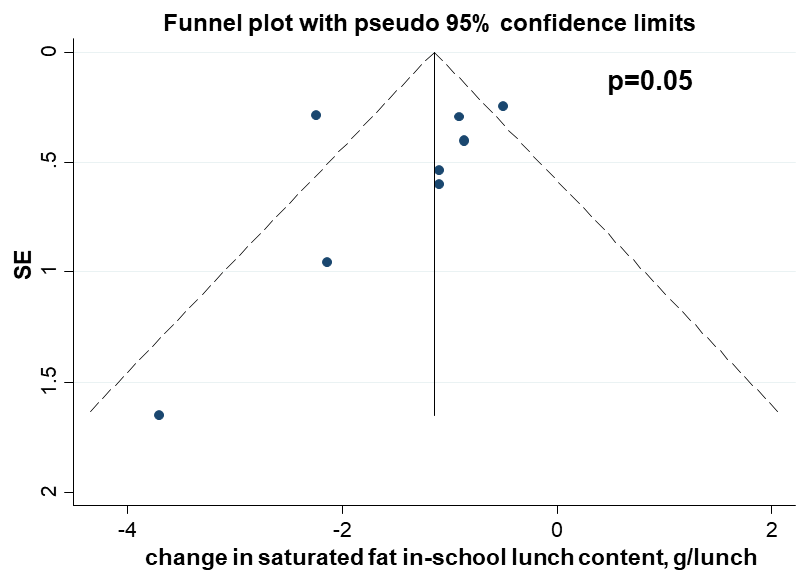


**Caloric intake Caloric content**


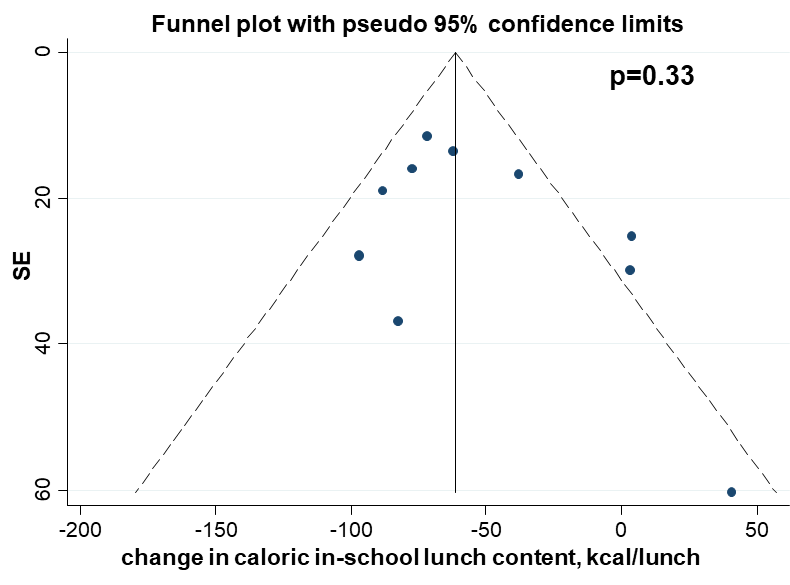

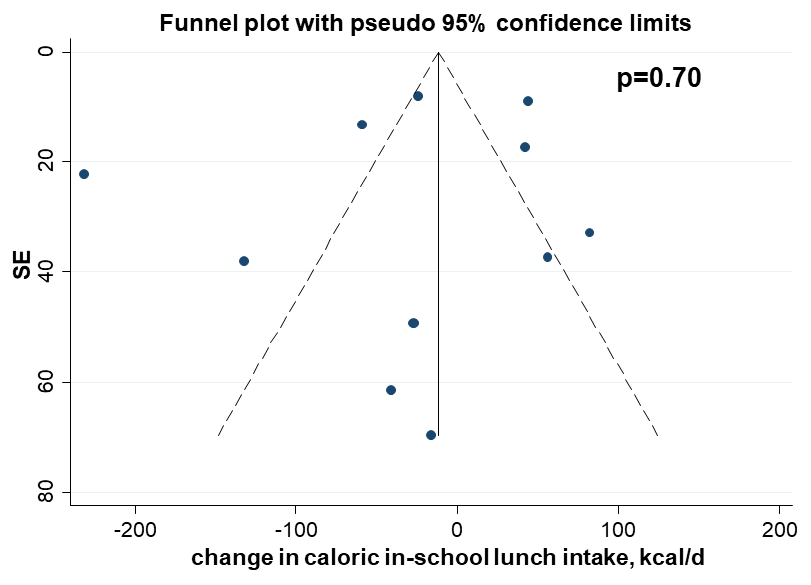


**Sodium intake**


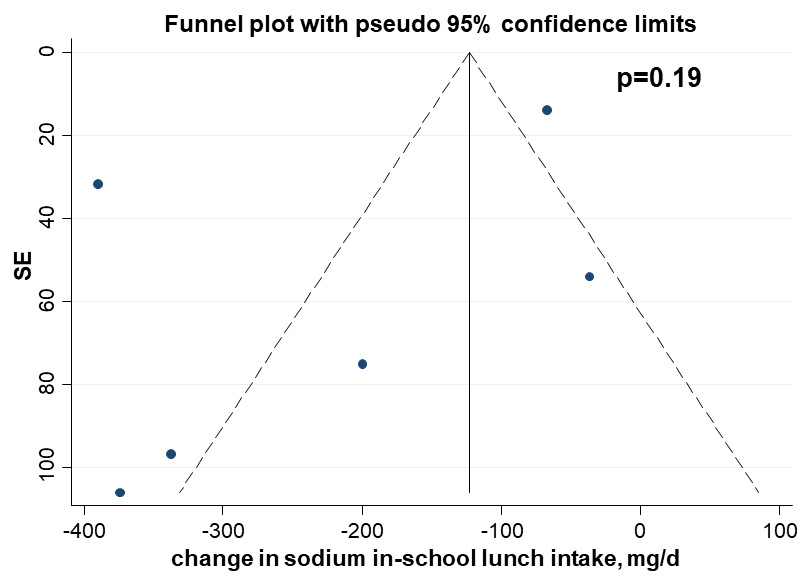


Figure Q. Begg’s funnel plots for graphical evaluation of potential publication bias for the effect of school meal standards on dietary intakes or meal contents in children. Intakes represent in-school lunch consumption. P-values based on the Begg’s adjusted rank-correlation test for presence of publication bias.

# **References**

1. Haroun D, Harper C, Wood L, Nelson M. The impact of the food-based and nutrient-based standards on lunchtime food and drink provision and consumption in primary schools in England. *Public Health Nutr.* 2011;14(2):209-218.

2. Hoppu U, Lehtisalo J, Kujala J, et al. The diet of adolescents can be improved by school intervention. *Public Health Nutr.* 2010;13(6A):973-979.
